# Mechanisms of far-red light-mediated dampening of defense against *Botrytis cinerea* in tomato leaves

**DOI:** 10.1093/plphys/kiab354

**Published:** 2021-07-24

**Authors:** Sarah Courbier, Basten L Snoek, Kaisa Kajala, Linge Li, Saskia C M van Wees, Ronald Pierik

**Affiliations:** 1 Plant Ecophysiology, Institute of Environmental Biology, Utrecht University, The Netherlands; 2 Theoretical Biology and Bioinformatics, Institute of Biodynamics and Biocomplexity, Utrecht University, The Netherlands; 3 Plant-Microbe Interactions, Institute of Environmental Biology, Utrecht University, The Netherlands

## Abstract

Plants detect neighboring competitors through a decrease in the ratio between red and far-red light (R:FR). This decreased R:FR is perceived by phytochrome photoreceptors and triggers shade avoidance responses such as shoot elongation and upward leaf movement (hyponasty). In addition to promoting elongation growth, low R:FR perception enhances plant susceptibility to pathogens: the growth–defense tradeoff. Although increased susceptibility in low R:FR has been studied for over a decade, the associated timing of molecular events is still unknown. Here, we studied the chronology of FR-induced susceptibility events in tomato (*Solanum lycopersicum*) plants pre-exposed to either white light (WL) or WL supplemented with FR light (WL+FR) prior to inoculation with the necrotrophic fungus *Botrytis cinerea* (*B.c.*). We monitored the leaf transcriptional changes over a 30-h time course upon infection and followed up with functional studies to identify mechanisms. We found that FR-induced susceptibility in tomato is linked to a general dampening of *B.c.-*responsive gene expression, and a delay in both pathogen recognition and jasmonic acid-mediated defense gene expression. In addition, we found that the supplemental FR-induced ethylene emissions affected plant immune responses under the WL+FR condition. This study improves our understanding of the growth–immunity tradeoff, while simultaneously providing leads to improve tomato resistance against pathogens in dense cropping systems.

## Teaser

The low red:far-red ratio enhances tomato susceptibility to the necrotrophic fungus *Botrytis cinerea* via delayed early pathogen detection and dampening of jasmonic acid-mediated defense activation.

## Introduction

Plants need sufficient light capture to sustain their photoautotrophic growth. At high planting densities, leaves grow closely and even overlap. This results in reduced light availability and changes in light quality due to preferential absorption of red (R, 600–700 nm) and blue light (B, 400–500 nm) and the reflection of far-red light (FR, ∼750 nm) toward neighboring plants in the canopy. Changes in the red: far-red ratio (R:FR) are sensed by a specialized family of photo-convertible photoreceptors known as phytochromes where phytochrome B (phyB) plays the major role in Arabidopsis (*Arabidopsis thaliana*; Franklin, 2008). A decrease of R:FR promotes the conversion of phyB from its active (Pfr) into its inactive (Pr) form. The photoinhibition of phyB by FR-enriched light leads to the release of PIF (PHYTOCHROME-INTERACTING FACTORS) transcription factors, which subsequently bind G and E boxes in the promoters of target genes to initiate shade avoidance-associated gene expression (Franklin, 2008; Lorrain et al., 2008; Franklin and Quail, 2010; Casal, 2013). PIF target genes are associated with auxin homeostasis and cell wall remodeling to control growth (Hornitschek et al., 2012; Li et al., 2012; Pedmale et al., 2016). Also, auxin biosynthesis and transport allow for coordination of growth responses across an organ or even the entire organism in response to heterogeneous light conditions (Kohnen et al., 2016; Pantazopoulou et al., 2017; Küpers et al., 2018). Low R:FR also promotes gibberellin (GA) biosynthesis via the induction of the GA biosynthesis genes *GA20ox and GA3ox* (reviewed in Ballaré and Pierik, 2017). GA binding to GIBBERELLIN INSENSITIVE DWARF1 (GID1) receptors initiates the ubiquitination and subsequent degradation of the negative growth regulators, DELLAs. Together, this results in PIF-mediated growth responses associated with rapid hypocotyl and petiole elongation as well as increased leaf angles (hyponasty) in Arabidopsis, characterized as the shade avoidance syndrome (SAS; Franklin, 2008; Casal, 2012).

Plant responses to (a)biotic stresses are also affected by low R:FR (Ballaré and Pierik, 2017; Courbier and Pierik, 2019). In Arabidopsis, additional FR radiation has been shown to enhance plant susceptibility toward an array of pathogens showing a strong interplay between light and defense signaling pathways. Low R:FR conditions negatively affect both salicylic acid (SA) and jasmonic acid (JA)-responsive gene expression, which are crucial hormonal pathways that induce downstream defense responses toward plant resistance (De Wit et al., 2013). Interplay between JA and the growth promoting hormone GA plays a role in compromised plant resistance in low R:FR conditions and this occurs, at least partly, via interaction between growth-inhibiting DELLA proteins and defense-suppressing JASMONATE-ZIM DOMAIN (JAZ) proteins. The low R:FR-induced increase in bioactive GA leads to reduced DELLA levels, thus releasing JAZ proteins that can suppress defense (Hou et al., 2010; Cerrudo et al., 2012; Yang et al., 2012). Low R:FR-induced downregulation of JA-mediated defense is further associated with an increased stability of JAZ10 (Leone et al., 2014; Cerrudo et al., 2017), and a decrease in bioactive JA levels in Arabidopsis (Fernández-Milmanda et al., 2020). These observations that low R:FR perception lead to growth promotion at the expense of defense responses is known as an integral part of the “growth-defense tradeoff”, which was originally thought to result from passive sink–source interactions and is now known to involve fine-tuned molecular control mechanisms in the plant (Campos et al., 2016; Ballaré and Austin, 2019; Major et al., 2020).

Light-dependent modulation of growth and susceptibility has mainly been studied in Arabidopsis, but has been less studied in other species, including tomato (*Solanum lycopersicum*). Low R:FR reduces tomato defenses against chewing and piercing insects (Izaguirre et al., 2006) and tomato *phyB1phyB2* double mutants exhibit increased leaf damage caused by *Mamestra brassicae* caterpillars compared to wild-type plants (Cortés et al., 2016). Interestingly, indirect defense responses are also promoted by phyB inactivation in tomato: a changed JA-regulated pool of volatile organic compounds attracted more *Macrolophus pygmaeus* predatory insects (Cortés et al., 2016). We observed recently that supplemental FR not only repressed resistance against herbivorous insects, but also against the necrotrophic pathogen *Botrytis cinerea* (*B.c.*) (Ji et al., 2019; Courbier et al., 2020). In fruiting tomato plants, supplemental FR has been shown to enhance growth, fruit set and dry mass partitioning toward tomato fruits while it also promoted foliar *B.c.* lesion development (Ji et al., 2019). We also recently demonstrated that supplemental FR leads to an increase in soluble sugars in tomato leaves responsible for increased lesion development induced by *B.c.* (Courbier et al., 2020). Nevertheless, little is known still about the timing of events involved in the supplemental FR–pathogen interaction and the associated transcriptome reprogramming.

*Botrytis cinerea* is one of the most destructive pathogens worldwide (Dean et al., 2012) and various transcriptome analyses have been performed on the Arabidopsis–*B.c.* pathosystem on both host and pathogen (Windram et al., 2012; Zhang et al., 2019; Soltis et al., 2020). Here, we aimed to unravel the effect of supplemental FR on the timing of hormonal and metabolic pathway transcriptional induction during *B.c.* infection of susceptible tomato plants that are often cultivated in dense stands. We performed a transcriptome analysis of a 30-h infection time course following a FR pretreatment to describe temporal changes of tomato in response to *B.c.* after a supplemental FR exposure. Our data show that supplemental FR pretreatment leads to a delay in *B.c.*-induced defense activation, by altering JA and possibly ethylene-dependent pathways. The transcriptome analysis highlighted a set of six *PROTEINASE INHIBITOR* (*PI*) genes that are induced only in WL-treated samples. Using these likely defense-associated genes as markers, we further resolved the roles of JA and ethylene in FR-induced susceptibility in tomato.

## Results

### FR-enriched light promotes shoot elongation and susceptibility toward *B.c.*

**Figure 1:**
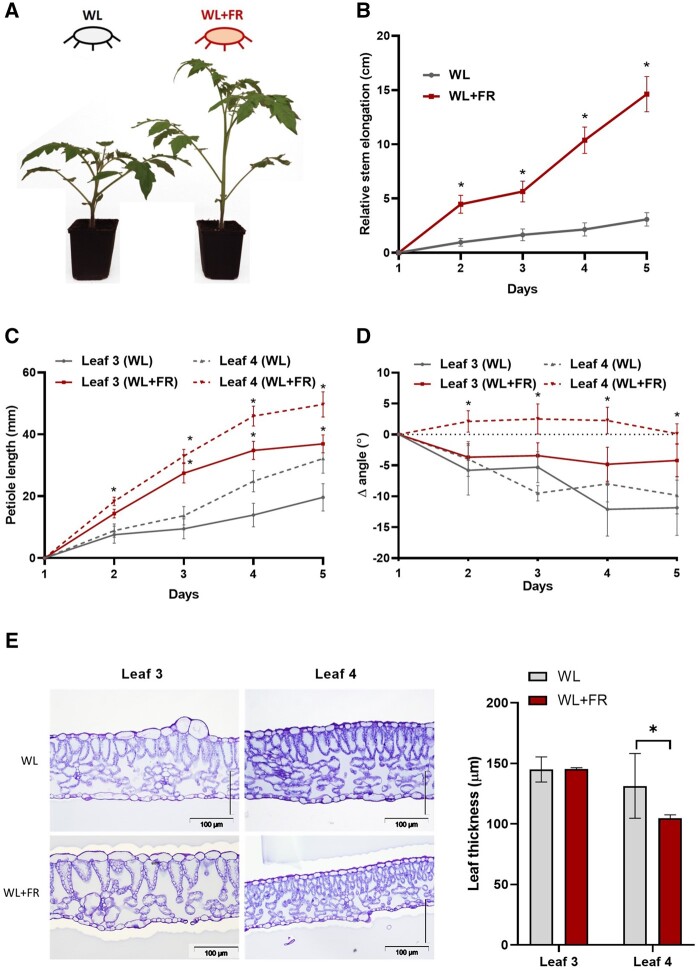
Supplemental FR light induces architectural changes in intact tomato plants. A, Four-week-old tomato plants photographed after 5 d in WL or WL+FR conditions. Stem elongation (B), petiole length (C), as well as leaf angle of the third (leaf 3) and fourth (leaf 4) oldest leaf (D) were recorded every day (ZT = 3). E, Thickness measurements performed on tomato leaflets exposed to either WL or WL+FR for 5 d. Measurements were performed on leaflets originating from a pre-existing leaf (leaf 3) prior to the start of the light treatment or newly formed (leaf 4) during the 5-d light treatment. Data show mean ±sem (Standard error of the mean) and asterisks represent significant differences between WL and WL+FR-exposed plants (and for leaf 3 and leaf 4 independently [C and D]) according to Student’s *t* test per timepoint (*P* < 0.05), *n* = 4–10.

**Figure 2:**
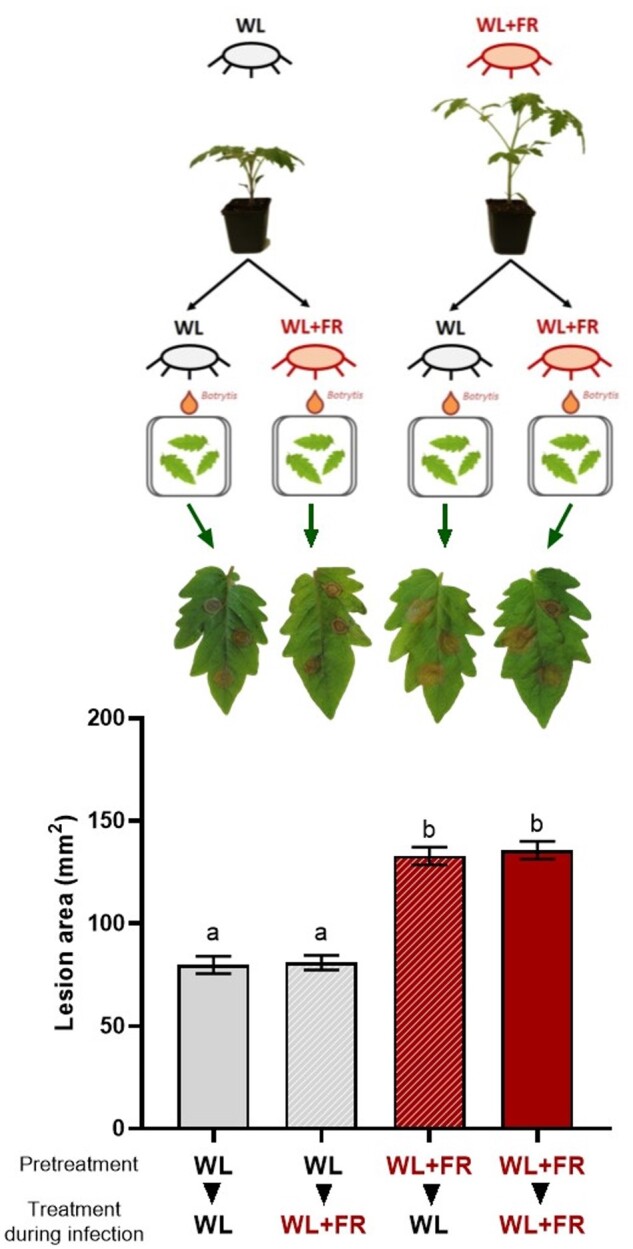
Supplemental FR light enhances tomato susceptibility toward *B.c.*. Leaflets originating from the third oldest leaf (leaf 3) of 4-week-old tomato plants were exposed to WL or WL+FR conditions for 5 d (pretreatment) prior to being detached, and drop-inoculated with *B.c.* spores. Inoculated detached leaflets were incubated in either WL or WL+FR for 3 d (treatment during infection). Images of the plants and leaves were digitally extracted for visual comparison. Plotted data represent the lesion area measured on the leaflets at 3 dpi. Data show mean ± sem and different letters represent significant differences between treatments according to analysis of variance (ANOVA), Tukey’s post hoc test (*P* < 0.05), *n* = 7–8 plants per treatment.

To investigate the morphological changes induced by FR light enrichment (WL+FR), 4-week-old intact tomato plants were exposed to either WL or WL+FR (Supplemental Figure S1) for 5 d and several growth parameters were recorded daily. Upon WL+FR exposure, plants exhibit a strong stem elongation compared to WL conditions (Figure 1, A and B). Although petiole length also increased upon WL+FR exposure (Figure 1C), plants did not show a hyponastic (upward leaf movement) response, a typical shade avoidance response in Arabidopsis (Pantazopoulou et al., 2017), but remained constant while the WL-treated plants exhibited some epinastic (downward) leaf movement over time (Figure 1D), probably because of leaf aging. In addition, tomato plants showed an increased lamina area and dry weight but no difference in specific leaf area (Supplemental Figure S2). Even though the leaf area increases slightly, the leaf thickness remained unchanged for leaves already formed before the start of the WL+FR treatment (leaf 3) while leaves that developed under WL+FR conditions (leaf 4) were slightly thinner than the WL control (Supplemental Figure S2A; Figure 1E). As leaf thickness could distort lesion area measurements, we focused our study on the third oldest leaf (leaf 3), which did not display changes in thickness. Then, we investigated the effect of FR supplementation applied before and/or during interaction with *B.c.* (Figure 2). The light pretreatment with WL or WL+FR was performed on whole plants before the first lateral leaflets from the third oldest leaf (leaf 3) were detached and inoculated with *B.c.* spores. Detached leaflets were chosen over whole plants to allow for inoculating a single leaflet multiple times, to optimize light capture and to keep high humidity during the inoculation to maximize disease success. The lesion area induced by the fungus was clearly larger on plants pretreated for 5 d with WL+FR before inoculation compared to WL-pretreated plants irrespective of the light treatment applied after inoculation (Figure 2). Although we used a standard bioassay on excised leaflets, we obtained similar findings in intact, whole plant systems (Supplemental Figure S3). Larger lesion size was associated with an increase in *B.c.* genomic DNA content in WL+FR-treated leaf tissue showing a positive effect of FR supplementation on *B.c.* growth in planta (Supplemental Figure S4A). We confirmed this in vitro by showing that *B.c.* mycelium biomass was increased by approximately 30% by WL+FR, even though the mycelium diameter on these plates remained unchanged (Supplemental Figure S4, B and C). Altogether, our results show that WL+FR exposure of tomato plants elicits strong tomato phenotype changes and enhanced performance of *B.c.* on these plants. As the outcome of the infection was not influenced by the light quality applied after inoculation, we conclude that a WL+FR pretreatment alters subsequent plant responses to *B.c.* and promotes its development in infected plant tissue.

### Gene expression dynamics are affected by supplemental FR and *B.c.*

**Figure 3:**
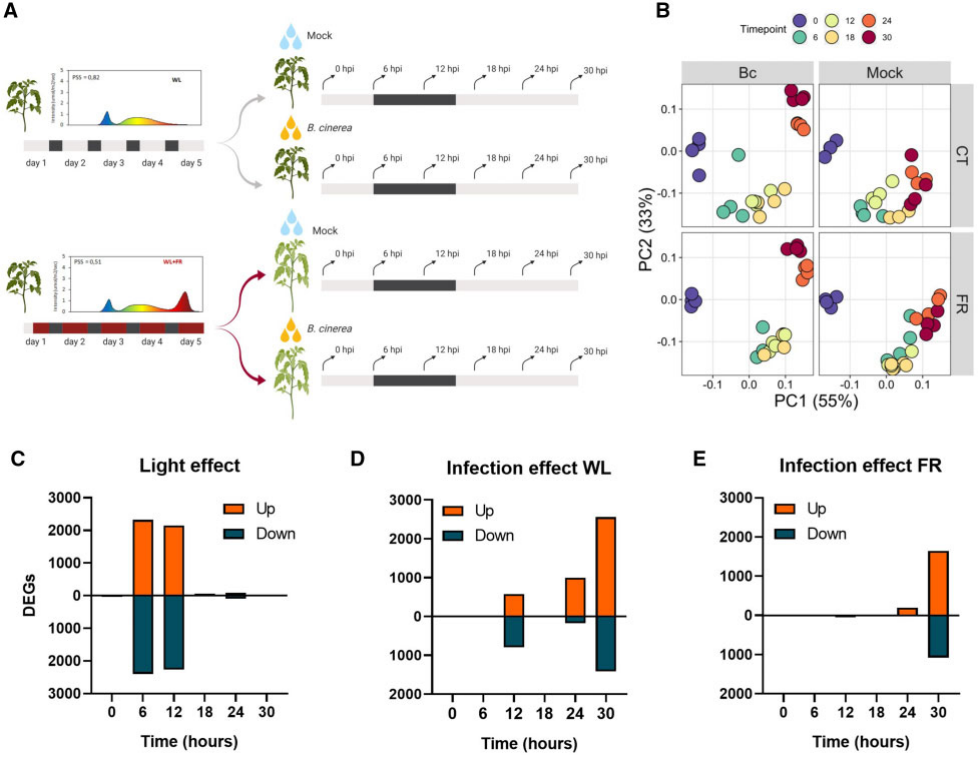
Responses to light and infection are not simultaneous and differ under WL+FR compared to WL conditions. A, Experimental set up and harvest time points for the time series of *B.c.* infection on tomato leaflets of WL- or WL+FR-pretreated plants. Four-week-old intact tomato plants exposed for 5 d in either the WL (gray) or WL+FR (red) condition prior to being detached and drop-inoculated with *B.c.* or treated with mock solution. Spectra represent the light background used as pretreatment light conditions. The dark gray area represents an 8-h dark period. After 5 d of light pretreatment, leaflets were detached and drop-inoculated with *B.c.* spores or a mock solution as a control and harvested at 0, 6, 12, 18, 24, and 30 hpi (hours post inoculation) represented by the arrows. The infected detached leaflets were incubated in WL conditions until harvested. B, Visualization of the variation in gene expression by PCoA. The analysis was separated per treatment namely WL, WL+FR, *B.c.* and Mock conditions. Different colors represent the different timepoints and each dot corresponds to a replicate (0, 6, 12, 18, 24, 30 hpi). C–E DEGs through time indicating upregulated (orange) and downregulated (dark blue) DEGs by comparing mock-treated samples in WL+FR compared to WL (C), *B.c.*-infected samples compared to mock samples after a WL (D) or WL+FR pretreatment (E) based on the Benjamini and Hochberg method for multiple testing adjusted *P* < 0.05.

We aimed to understand the mechanisms underlying this WL+FR-induced susceptibility in tomato by investigating the associated transcriptome responses. Expression data were obtained from leaf tissue (originating from leaf 3) pre-exposed to WL or WL+FR prior to inoculation with *B.c.* spores or with a mock solution on detached leaflets (Figure 3A). During the infection, all detached leaflets were placed under WL conditions, excluding direct growth-promoting effects of FR on *B.c.* itself (Supplemental Figure S4, B and C), and thus allowing us to study the plant responses specifically. The experiment consisted of 95 samples collected over two pretreatments performed on intact plants (WL and WL+FR light), two treatments performed on detached leaflets (mock versus infection with *B.c.*), six time points and three to four replicates per sample (Figure 3A;Supplemental Data Set S1). We first performed a principal coordinate analysis (PCoA) where the mapped reads segregated according to the light treatment at 6 and 12 hpi and the effect of the infection became visible at the later timepoint (24 and 30 hpi; Figure 3B). These dynamics also reflected the number of differentially expressed genes (DEGs) where thousands of genes were modulated upon WL+FR pre-exposure at 6 and 12 hpi and upon infection at later timepoints (Figure 3, C–E). At 0 hpi, only few genes were modulated by WL+FR exposure (15 up- and 23 downregulated genes), implying that light quality does not extensively influence the tomato transcriptome after an extended period of time but rather upon reexposure to WL after inoculation (Figure 3C). This was further confirmed by reverse transcription quantitative polymerase chain reaction (RT-qPCR) on WL+FR-regulated genes which were induced at the start of the FR enrichment but only slightly after several days (Supplemental Figure S5). On the contrary, *B.c.*-responsive genes are modulated at later time points, likely resulting from a lag phase between the inoculation and the actual infection of leaf cells (Figure 3, D and E). Importantly, *B.c.*-responsive genes are detected from 12 hpi onward in WL-pretreated samples while these are detected only from 24 hpi onward in WL+FR-pretreated samples, suggesting a substantial delay in defense gene activation (Figure 3, D and E, respectively). In addition, many more genes were modulated by *B.c.* in WL-pretreated samples (6,511 DEGs) compared to WL+FR-pretreated samples (2,944 DEGs). Altogether, these observations indicate drastic FR-mediated dampening of the *B.c.*-responsive transcriptome response.

### Light impacts gene expression at early timepoints

**Figure 4:**
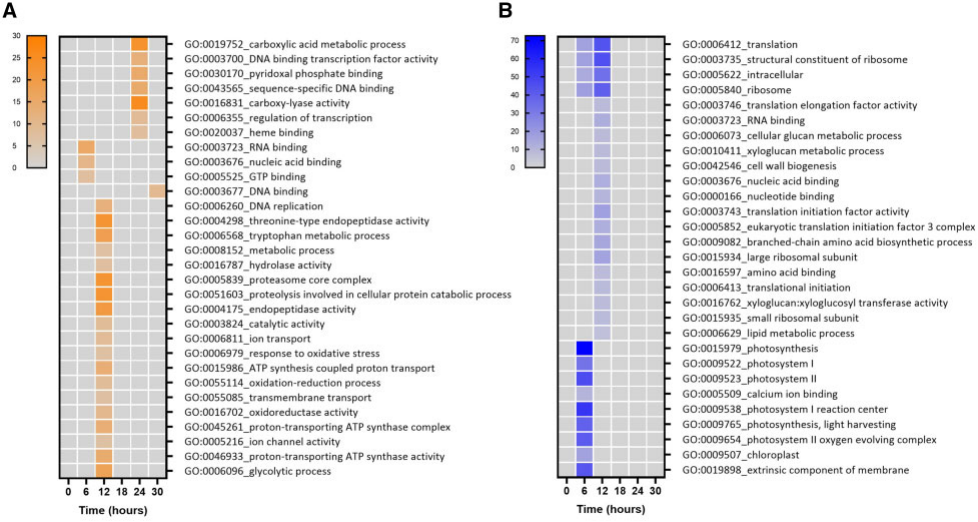
GO analysis of DEGs in the WL+FR pretreatment compared to the WL pretreatment under noninfected conditions. Heatmap of the GO categories based on –log_10_*P*-value where shades of orange represent enrichment in upregulated DEGs (A) and blue in the downregulated DEGs (B). Comparisons were performed between WL+FR and WL-pretreated samples (considering mock-inoculated samples only) for each timepoint (0, 6, 12, 18, 24, and 30 h).

**Figure 5:**
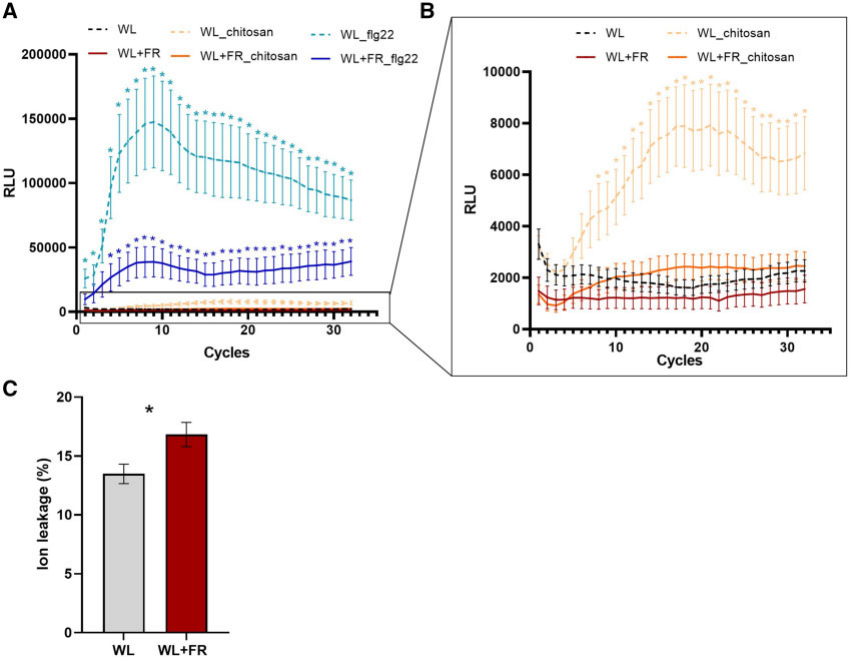
Supplemental FR pre-exposure alters ROS production and cell integrity. A, B, ROS quantification by luminescence on leaf discs originating from 4-week old intact tomato plants treated for 5 d in WL or WL+FR light conditions. Leaf discs were exposed to either the bacterial elicitor flagellin (_flg22), the fungal elicitor chitosan (_chitosan), or were left unelicited and taken as controls. Data represent mean ± sem and each measurement (cycle) lasted approximately 100 s. *n* = 8. Asterisks represent significant difference according to Student’s *t* test (*P* < 0.05) between elicited samples and their respective unelicited controls (WL or WL+FR), *n* = 12. C, Electrolyte leakage quantification of tomato leaf discs originating from 4-week-old intact tomato plants exposed for 5 d to WL or WL+FR light conditions. Data represent mean ± sem. Asterisk represents significant difference according to Student’s *t* test (*P* < 0.05), *n* = 5.

To investigate the direct effect of light quality on gene expression over time, we selected DEGs affected by the WL+FR pre-exposure in the absence of the pathogen. Most of the supplemental FR-mediated gene expression changes were observed at 6 and 12 h after leaf excission and return to WL (Figure 3, B and C). By performing a gene ontology (GO) term enrichment on all WL+FR-responsive genes for each timepoint, we observed the upregulation of GO categories related with oxidoreduction processes, regulation of transcription, proteasome activity, and glycolytic process, indicating an effect of WL+FR pre-exposure on gene expression and metabolism through time (Figure 4A). At the same time we noticed a downregulation of glucan- and cell biogenesis-related processes upon WL+FR pretreatment at 12 hpi (Figure 4B). The oxidoreduction GO term enrichment could hint at regulation of oxidative stress responses upon pathogen detection by supplemental FR and, therefore, we performed a luminol-based hydrogen peroxide (H_2_O_2_) quantification. Plants were elicited with the fungal elicitor chitosan or with the bacterial elicitor flagellin (flg22) to initiate a reactive oxygen species (ROS) burst. A strong and rapid increase in H_2_O_2_ production occurred upon elicitation with both elicitors (Figure 5, A and B), but this burst was severely suppressed in WL+FR-pretreated leaf tissue (Figure 5A). Based especially on the GO enrichments found for downregulated genes (Figure 4B), we tested whether supplemental FR could affect cell wall and/or membrane integrity. By performing an electrolyte leakage assay, we observed an increase in the percentage of electrolyte loss in the WL+FR-pretreated plants compared to WL-pretreated plants (Figure 5C), which indicates a reduced membrane integrity caused by WL+FR pretreatment that could in principle promote pathogen-induced cell death.

### Supplemental FR light delays pathogen recognition and dampens defense activation

**Figure 6:**
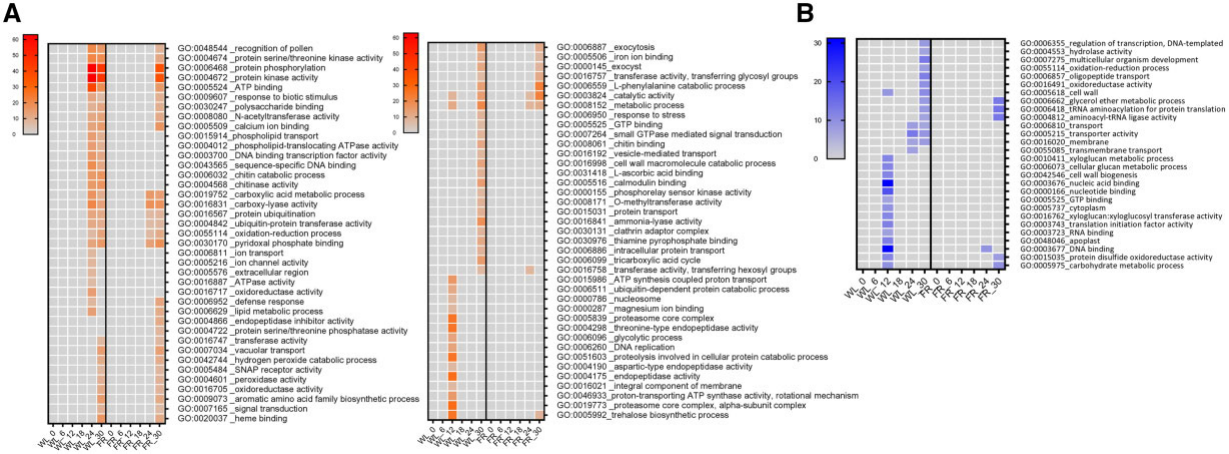
GO analysis of *B.c.*-responsive genes. Heatmap of the GO based on (–)log_10_(*P*-value) of *B.c.-*responsive DEGs in WL and WL+FR light exposed plants where orange represents enrichment in upregulated DEGs (A) and blue in downregulated DEGs (B). Comparisons were performed between *B.c.*-infected samples and mock-inoculated samples for each timepoint (0, 6, 12, 18, 24, and 30 h) in WL and WL+FR-pretreated samples (WL and FR), respectively.

As *B.c.*-responsive gene modulation was delayed by WL+FR (Figure 3, D and E), we tested all *B.c.*-responsive genes for GO term enrichment (Figure 6). Out of 76 enriched processes found upregulated upon *B.c.* infection in WL-pretreated samples, only 34 were shared between WL and WL+FR-treated samples. From these 34 GO categories shared between both light pre-treatments, 14 categories were delayed by WL+FR light (Figure 6A). At 12 hpi, we observed a striking inhibition of GO categories associated with protein catabolic processes (GO:0006511 and GO:0051603) via the proteasome (GO:0005839 and GO:0019773) or endopeptidase activity (GO:0004175, GO:0004190, and GO:0004298) by WL+FR (Figure 6A). At later timepoints, we found enrichment of GO terms associated with chitin binding (GO:0008061) and chitin degradation processes (GO:0006032 and GO:0004568) in WL but not in WL+FR-pretreated samples. This could suggest that supplemental FR inhibits the plant response to degrade the fungal cell wall. Consistently, genes associated with responses to biotic stimulus or defense response upregulated at 24 hpi in WL, are absent or delayed in WL+FR (Figure 6A). These observations fit the notion that WL-pretreated plants recognize the pathogen and actively restrict its progression better than WL+FR-pretreated plants. At 24 and 30 hpi in WL+FR-pretreated samples, we also observed a delay in the upregulation of genes associated with protein phosphorylation (GO:0006468) and protein kinase activity (GO:0004674 and GO:0004672), possibly involved in downstream defense responses. DNA binding transcription factor activity (GO:0003700) was upregulated in WL-pretreated samples at 24 hpi but absent in WL+FR-pretreated samples. This category was composed of genes encoding WRKY transcription factors of which some might be involved in SA and JA signaling (Pandey and Somssich, 2009) and ETHYLENE-RESPONSIVE FACTORS (ERFs) mainly regulated by ethylene (Müller and Munné-Bosch, 2015).

**Figure 7:**
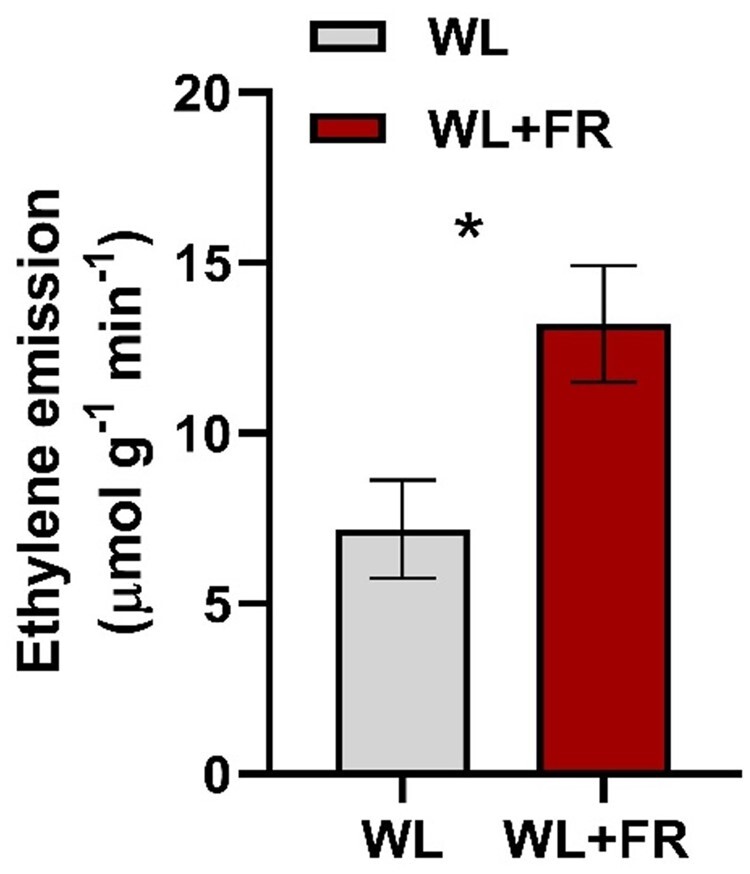
Leaf ethylene emissions are increase by supplemental FR. Ethylene emission quantified after 5 d of WL and WL+FR light treatments on whole tomato plants. Ethylene levels were measured on detached leaflets (originating from the third oldest leaf) incubated for 30 min in air tight plastic syringes. *n* = 6–8. Data represent mean ± sem. Asterisk represents significant difference according to Student’s *t* test (*P* < 0.05).

The involvement of ethylene was confirmed by an upregulation of ethylene biosynthesis genes *1-AMINOCYCLOPROPANE-1-CARBOXYLIC ACID OXIDASE* (*ACO*) and *SYNTHASE* (*ACS*) at 24 and 30 hpi in WL and only at 30 hpi in WL+FR-pretreated plants (associated with GO:0003824 and GO:0055114 in Figure 6A). Despite the delayed induction of ethylene biosynthesis genes upon infection with *B.c.* in WL+FR-pretreated plants, ethylene emissions were elevated by WL+FR as compared to WL (Figure 7); a classic effect of supplemental FR (Kegge and Pierik, 2010). Altogether, our data indicate that supplemental FR affects both ethylene and JA signaling in tomato defense against *B.c.*. Upon infection, only 6 out of 28 downregulated categories found in WL-pretreated samples were shared with WL+FR-pretreated samples (Figure 6B). Interestingly, GO categories associated with xyloglucan and cellular glucan metabolism as well as xyloglucan:xyloglucosyl transferase activity and cell wall biogenesis (GO:0010411, GO:0006073, GO:0016762, and GO:0042546) were strongly downregulated at 12 hpi in WL conditions only (Figure 6B). The downregulation of glucan-related structural processes could potentially reflect carbohydrate reallocation toward energy production (e.g. GO:0015986 and GO:0046933; Figure 6A) to respond to the attacker. Interestingly, we did not observe such phenomenon in WL+FR-pretreated plants probably as the fungal response is not fully amplified to the level of detection at 12 hpi. Combined with previous research showing that WL+FR-pretreated plants display elevated soluble sugar levels in leaves promoting disease severity (Courbier et al., 2020), the delay in transcriptome changes in WL+FR-pretreated plants could be an additional explanation for the increased susceptibility of these plants. Taken together, our results indicate that *B.c.* infection triggers strong transcriptome reprogramming in WL which is substantially dampened, delayed, or nonexistent in WL+FR-pretreated samples, and likely associated with increased susceptibility.

### Supplemental FR-induced susceptibility coincides with downregulation of hormonal signaling

**Figure 8:**
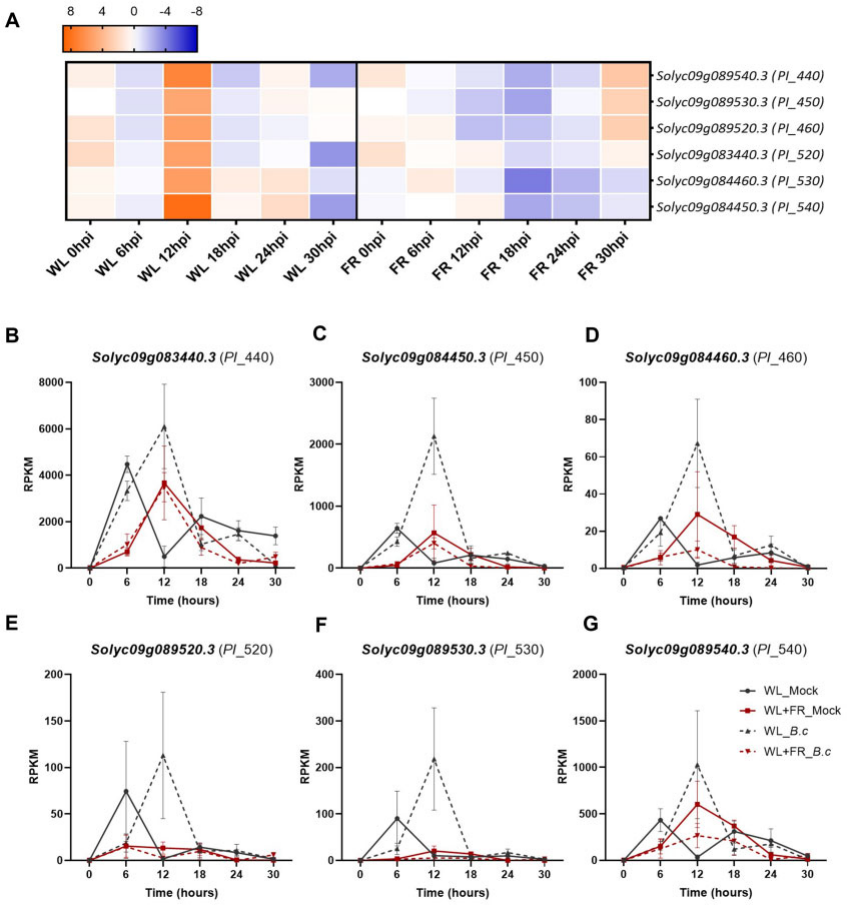
Dynamics of expression of *PI* genes significantly interacting in the three-way light*infection*time ANOVA. A, Heatmap corresponding to the log_2_FC of six *PI* genes in response to *B.c.* infection after a 5-d WL or WL+FR pretreatment at 0, 6, 12, 18, 24, and 30 hpi. Orange and blue colors represent up- and downregulation, respectively. B–G, Expression patterns (RPKM) of the six *PI* genes. Each plot corresponds to the expression patterns of an individual gene through time in the four conditions tested, namely WL (gray) or WL+FR (red) pre-exposed plants inoculated with *B.c.* (dashed lines) or treated with a mock solution (plain lines). Plotted data represent the mean of replicates per timepoints ± sem.

To define the core gene expression module associated with FR-induced susceptibility through time in tomato, we performed a three-way ANOVA on light*infection*time to select genes interactively modulated by light, infection and time (Supplemental Figure S6). We then ran a GO enrichment analysis on this group of genes and identified 11 significantly over-represented categories (Table 1;Supplemental Dataset S2). One of these categories was associated with response to wounding (GO:0009611) and was composed of six genes, all encoding PIs. These six genes were upregulated at 12 hpi upon *B.c.* infection in WL-pretreated samples only and not in the WL+FR-pretreated samples (Figure 8A). *PI* genes are JA-responsive genes that are highly induced upon *B.c.* infection in tomato (El Oirdi et al., 2011), and their differential regulation between WL and WL+FR-pretreated leaves, therefore, suggests involvement of JA in the FR-induced susceptibility and a possible alteration of the JA-mediated *PI* gene induction by WL+FR (Figure 8A). RPKM-normalized expression profiles for these six genes in all conditions tested in the RNA-seq data showed that all *PI* genes seem to be transiently upregulated in response to *B.c.* in WL conditions while this induction was strongly dampened in WL+FR conditions (Figure 8, B–G). Interestingly, we observed a peak of expression of all genes at 6 hpi in WL-treated samples in the absence of *B.c.* possibly reflecting the wounding responses triggered by detaching the leaflets from the plants prior to the bioassays. In WL+FR conditions, this putative wound-mediated *PI* peak was either delayed (Figure 8, B–D, G) or absent (Figure 8, E and F). Altogether, these results show that *PI* genes are responsive to *B.c.* infection (and possibly wounding) and their expression is dampened and/or delayed by WL+FR.

**Table 1.** GO categories enriched in DEGs from the light*infection*time interaction.

GO identifier	Description	Genes	*P*-value
GO:0009611	Response to wounding	6	3.68958E-06
GO:0008171	O-methyltransferase activity	6	2.8157E-05
GO:0008152	Metabolic process	52	5.53184E-05
GO:0006629	Lipid metabolic process	17	5.56319E-05
GO:0004867	Serine-type endopeptidase inhibitor activity	6	6.22373E-05
GO:0004866	Endopeptidase inhibitor activity	4	0.000447478
GO:0043565	Sequence-specific DNA binding	21	0.002891596
GO:0004190	Aspartic-type endopeptidase activity	9	0.004355757
GO:0016747	Transferase activity, transferring acyl groups other than amino-acyl groups	9	0.00592484
GO:0030170	Pyridoxal phosphate binding	10	0.006857719
GO:0006869	Lipid transport	4	0.008920639
GO:0016627	Oxidoreductase activity, acting on the CH–CH group of donors	4	0.008920639

### Supplemental FR delays activation of JA signaling.

**Figure 9:**
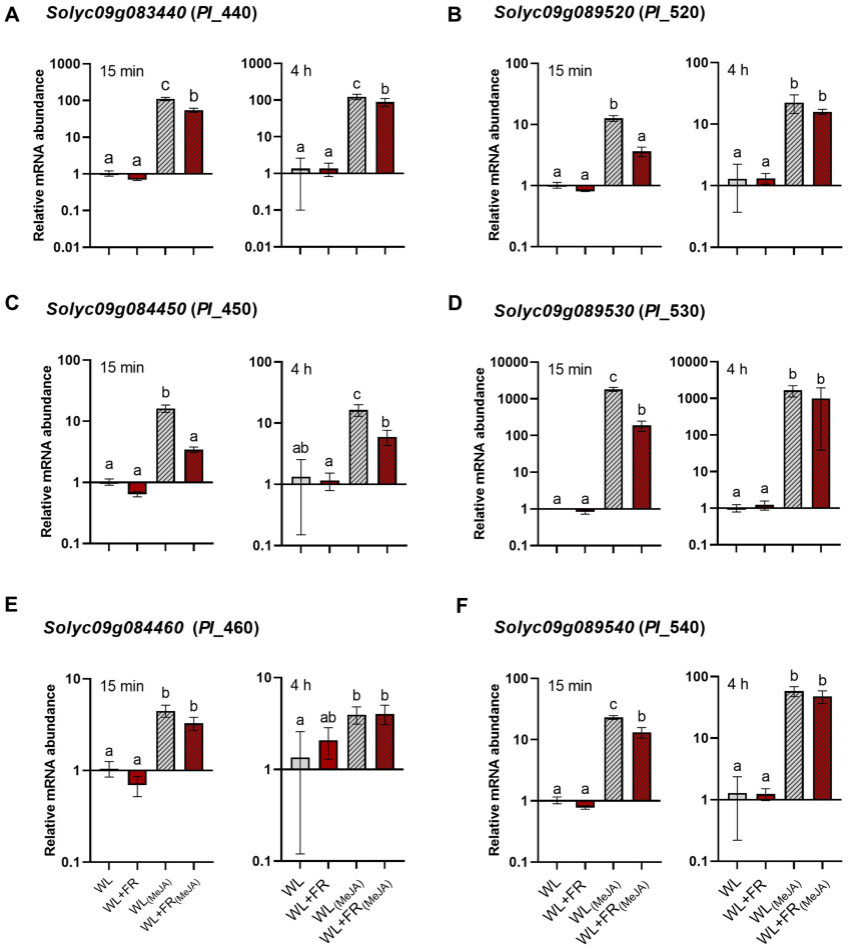
Supplemental FR causes a delay in the JA-mediated response in whole tomato plants. A–F, Transcript abundance analysis for six *PI* genes after a WL (gray) or WL+FR (red) pretreatment followed by an exogenous MeJA treatment (100 µM; dashed bars) or a mock solution (plain bars) on intact tomato plants. Leaf material was harvested from the third oldest leaf of whole tomato plants at 15 min and 4 h after the start of the MeJA treatment. Expression data are relative to WL conditions for each timepoint. Data represent mean ± sem. Letters represent significant differences according to ANOVA, Tukey’s post hoc test (*P* < 0.05).

To confirm the trends of *PI* gene expression (Figure 8, B–G), and further study the involvement of JA in the regulation of these genes, we sprayed the third leaf of intact tomato plants with 100-µM methyl-jasmonate (MeJA) or a mock solution and quantified mRNA abundance 15 min and 4 h later (Figure 9). After 15 min, we observed a strong induction of *PI* genes under WL conditions, which was significantly reduced in WL+FR conditions for five out of the six *PI* genes (Figure 9, A–D, F). After 4 h, there was still a significant reduction due to WL+FR in MeJA-induced expression levels of two of the *PI* genes (Figure 9, A and C) but for the other four *PI* genes the induction level by MeJA was comparable between WL- and WL+FR-pretreated samples (Figure 9, B, D–F). This suggests a delay rather than an overall inhibition in the induction of *PI* genes by MeJA under WL+FR enrichment.

### Supplemental FR interacts with JA and ethylene pathways to affect disease development

**Figure 10:**
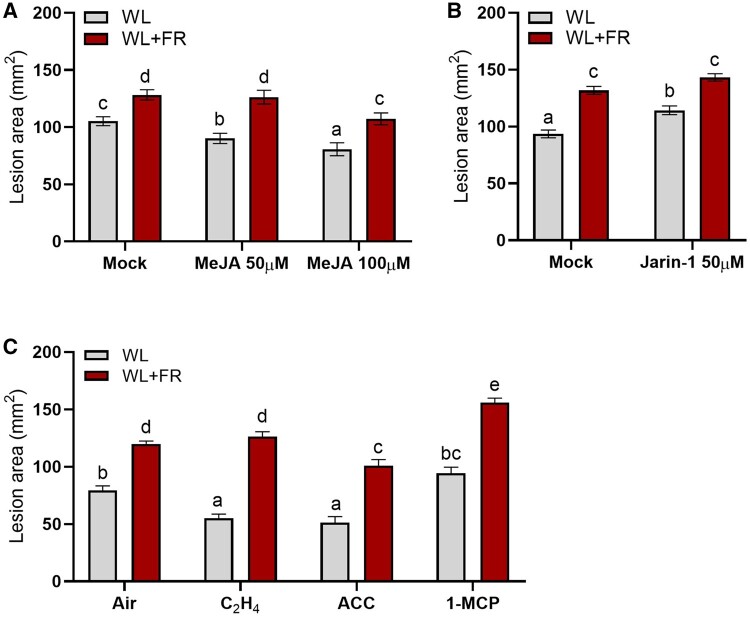
FR enrichment affects ethylene and JA-mediated defense in tomato toward *B.c..* Disease rating on tomato leaflets after 5 d of WL and WL+FR light treatments followed by an exogenous MeJA (50 µM and 100 µM) treatment (A), a Jarin-1 (JA biosynthesis inhibitor; 50 µM) treatment (B) or exposure to either ethylene (C_2_H_4_), the ethylene precursor ACC, or the ethylene receptor blocker 1-MCP treatment (C) prior to inoculation in WL. The 5-d light pretreatment (WL or WL+FR) was applied on whole plants prior to inoculation on detached leaflets. *n* = 7–8 plants per treatment. Data represent mean ± sem. Different letters represent significant differences according to ANOVA, Tukey’s post hoc test (*P* < 0.05).

As defense responses against necrotrophic pathogens are mainly regulated by JA and ethylene (Penninckx et al., 1998; Du et al., 2017), we assessed the involvement of these two hormones in tomato resistance toward *B.c.* and its modulation by supplemental FR. First, we tested the effect of exogenous MeJA (50 µM and 100 µM) and the JA biosynthesis inhibitor Jarin-1, which inhibits the activity of JASMONATE RESISTANT1 (JAR1) catalyzing the conversion of (+)-7-iso-JA into JA-Ile (Meesters et al., 2014; Figure 10, A and B). As expected, exogenous MeJA could enhance plant resistance in WL and this effect was stronger at increased MeJA concentrations confirming that JA is promoting defense (Figure 10A). Although MeJA could also partly enhance the resistance in WL+FR-treated plants, the effect was only visible at the highest MeJA concentration applied, indicating that WL+FR-pretreated plants had reduced responsiveness to MeJA as compared to WL plants. The addition of Jarin-1 (50 µM) increased plant susceptibility to *B.c.* in WL-treated leaflets. However, it did not significantly affect the resistance of WL+FR-pretreated tissue as the lesion size upon Jarin-1 treatment was similar to the mock conditions (Figure 10B). Altogether, these JA manipulations explain some of the WL+FR effects, but do not explain the full effect, implying other layers of resistance that can be altered by WL+FR. Since JA often acts together with ethylene, we also performed bioassays on detached leaflets treated with air (as a control), ACC (ethylene precursor), ethylene (C_2_H_4_), or 1-MCP (ethylene perception inhibitor) prior to inoculation with *B.c.* spores (Figure 10C). Ethylene improved resistance in WL-treated plants, and ACC application improved resistance in both WL- and WL+FR-pretreated plant tissue. In addition, 1-MCP had minor effects in WL-pretreated leaflets while it further compromised tomato resistance in WL+FR-treated plants in line with the protective effect of ethylene in tomato resistance against *B.c.* (Díaz et al., 2002; Figure 10C). These data confirm that ethylene promotes resistance against *B.c.* in tomato, and indicate that the elevated ethylene emissions in WL+FR partly counterbalance the WL+FR-induced increased disease development upon *B.c.* infection.

## Discussion

Here, we studied the transcriptome dynamics in WL and WL+FR-pretreated tomato plants upon infection with *B.c.* and unraveled multiple potential processes that could underlie the FR-induced susceptibility in tomato. Collectively, our findings suggest that WL+FR dampens metabolic, defense-, and hormone-related processes involved in adequate plant defense against *B.c.* infection. It was previously shown that a shade avoidance phenotype, for example in the cryptochrome 1 (*cry1*) mutant, per se is not enough to enhance plant susceptibility (Cerrudo et al., 2012), whilst inhibition of shade avoidance in the shade avoidance mutants *sav3-2* (still able to sense FR light) could not prevent the increased susceptibility under supplemental FR (Moreno et al., 2009). These studies indicate the involvement of FR-dependent signaling routes rather than passive tradeoffs. The inhibition of defense by FR light is set in motion especially upon prolonged supplemental FR exposure prior to infection with *B.c.* in tomato (Courbier et al., 2020). Here, we show how this severely delays the molecular response of tomato upon inoculation with *B.c.* partly via a reduced JA response.

### Control of cell wall biogenesis and ROS-mediated plant defense by WL+FR

The cell wall is one of the first layers that pathogens encounter upon infection. The thickness and permeability of the cell wall and cell membrane might even be key to control pathogen penetration success (Underwood, 2012). In our conditions, we observed a downregulation of cell wall biogenesis upon FR exposure as well as upon *B.c.* infection and an increase in electrolyte leakage in WL+FR-pretreated plants compared to WL-pretreated plants (Figures 4B, 5C, and 6B) that could potentially facilitate pathogen penetration in plant tissue. Upon pathogen attack, the production of ROS by plants is essential for the establishment of resistance (Chinchilla et al., 2007) and we observed a clear upregulation of genes associated with oxidoreduction processes in WL+FR-pretreated samples compared to WL (Figures 4A and 6A). Although genes associated with oxidoreduction processes are upregulated by supplemental FR, it is difficult to assess precisely whether WL+FR promotes oxidase or reductase activity. However, physiological data demonstrated that WL+FR-pretreated plants had a much weaker H_2_O_2_ production burst upon elicitation with flagellin and chitosan (Figure 5, A and B). It is possible that the strong upregulation of the genes associated with oxidoreduction processes could lead to a redox unbalance where plants cannot produce ROS as efficiently as in WL. However, we cannot exclude the possibility that WL+FR-pretreated plants over-scavenge ROS resulting in weaker ROS-mediated defense responses.

### Supplemental FR delays pathogen detection and activation of defense signaling

Plants are able to recognize microbe-associated conserved motifs known as MAMPs at the plasma membrane as a very early signal preceding downstream defense gene activation (Jones and Dangl, 2006). Chitin is the main component of fungal cell wall and constitutes a very efficient MAMP that can trigger immediate defense gene activation upon a pathogen challenge (Zipfel, 2014). WL-pretreated plants showed an upregulation of chitin binding- and chitin degradation-associated processes upon *B.c.* infection (GO:0006032 and GO:0004568), whereas WL+FR-pretreated plants did not (Figure 6A). This is in line with the dampened chitosan-induced ROS burst in WL+FR-treated plants shown in Figure 5B. Also, GO categories associated with “protein serine/threonine kinase activity”, “protein phosphorylation”, and “protein kinase activity” that are enriched in upregulated DEGs at 24 and 30 hpi in *B.c.*-challenged WL-pretreated plants are present only at 30 hpi in WL+FR-pretreated plants (Figure 6A). Most of the DEGs associated with these three categories encode receptor like kinases (RLKs), leucine-rich repeat RLK (LRR-RLKs), and mitogen-activated protein kinases (MAPK). These are gene families of which members are often associated with pathogen detection at the plasma membrane and the subsequent defense gene activation (Zipfel, 2014). Interestingly, we also found *WALL-ASSOCATED_KINASE (WAK)-LIKE KINASE* (*Solyc02g090970*) upregulated in WL-treated plants only. As WAK1 has been identified as a galacturonides receptor that promotes *B.c.* resistance in Arabidopsis (Brutus et al., 2010), the lack of *Solyc02g090970* induction in supplemental FR would be consistent with the increased disease susceptibility and could indicate a putative delay in pathogen detection at the cell surface. This could in turn delay the induction of chitinases (Figure 6A) that degrade the fungal cell wall, which would indirectly promote pathogen colonization in supplemental FR-pretreated leaf tissue compared to WL. In addition, a delayed pathogen detection at the cell surface correlates with a reduction of subsequent defense signaling, which is supported by a delayed representation of the GO categories associated to “defense response” and “response to biotic stimulus” in the supplemental FR-pretreated plants challenged with *B.c.* (Figure 6A). Altogether, these observations indicate a clear effect of supplemental FR on the response to infection, possibly due to a delay in pathogen recognition at the membrane that could result in a delay in chitin degradation and defense gene activation through the MAMP-mediated defense signalling cascade.

### Several JA-induced *PI* genes are suppressed by WL+FR

The three-way ANOVA light*infection*time analysis revealed the core gene expression patterns associated with FR-induced susceptibility. Out of the 131 genes significant for the interaction, we found six *PI* genes highly induced upon infection by *B.c.* at 12 hpi in WL and not in WL+FR-pretreated samples (Figure 8A;Supplemental Figure S6). *PI* genes have previously been reported to be strongly induced upon *B.c.* infection and promote plant resistance in a JA-dependent manner (El Oirdi et al., 2011). In our data, we observed that exogenous MeJA treatment induced *PI* genes within 15 min and that the level of expression was reduced in WL+FR-treated plants compared to WL. However, *PI* expression reached similar levels between WL and WL+FR-exposed plants after 4 h of MeJA treatment showing either a reduced JA sensitivity or a delay in gene induction by supplemental FR-pretreatment (Figure 9, C–H). In addition, tomato resistance against *B.c.* was promoted by exogenous MeJA treatment while it was compromised by Jarin-1 in WL-treated plants (Figure 10, A and B). However, we could only rescue resistance in WL+FR samples at the highest applied concentration of 100-µM MeJA, while 50-µM already promoted resistance in WL-treated plants confirming the hypothesis of a lower JA sensitivity in WL+FR (Moreno et al., 2009; Cerrudo et al., 2012; Izaguirre et al., 2013; De Wit et al., 2013). As supplemental FR appears to reduce JA responsiveness (Figure 9), it is likely that plants do not respond to JA-inducing *B.c.* infection as effectively as WL-exposed plants do, therefore allowing the pathogen to develop faster on leaves treated with supplemental FR (Supplemental Figure S3A). In addition, we showed that the susceptibility of WL+FR-pretreated leaflets was always higher than WL-treated leaflets, even upon high concentration of exogenous MeJA, pointing toward other layers of direct or indirect immunity. We showed previously that WL+FR-treated tomato leaves contain elevated soluble sugar levels, which promoted the proliferation of *B.c.* (Courbier et al., 2020). Although this increased sugar accumulation was partially JA-dependent, it could not be fully explained by JA variations, and thus constitutes a partially independent pathway of supplemental FR-induced susceptibility (Courbier et al., 2020).

### Ethylene signaling is affected by supplemental FR

Among the GO processes and genes modulated upon infection, genes encoding ERF transcription factors were only upregulated in WL-pretreated samples and genes encoding ethylene biosynthesis enzymes were induced earlier in WL-pretreated plants compared to WL+FR upon infection with *B.c.* (Figure 6A). Since some ERF proteins are important for defense against *B.c.*, as was shown for ERF3.C3 in tomato (Du et al., 2017), this delay is consistent with reduced activation of the pathogen response pathway. We also observed that exogenous ACC or ethylene treatment promoted plant resistance to the fungus (Díaz et al., 2002; Figure 7). The promoting effect of ethylene alone on plant resistance was weak in WL+FR-pretreated plants, as compared to ACC. We expect that ACC continues to be converted into ethylene by the leaflets and therefore accumulates in the sealed petri dishes giving a long-lasting ethylene treatment compared to ethylene treatment alone, which is dissipated within minutes after opening the desiccators. Since ethylene is beneficial for plant defense, it is paradoxical that supplemental FR light would simultaneously promote ethylene emission, thus promoting resistance, while at the same time enhancing susceptibility through dampening of the JA pathway. Apparently, under low R:FR conditions, the elevated ethylene emissions exert some control over the enhanced susceptibility to pathogens. Indeed, when inhibiting the plant’s ethylene receptors with 1-MCP, disease development in the low R:FR-exposed plants became even more severe. We, therefore, conclude that under low R:FR conditions indicating proximate neighbors, plants downregulate the partially JA-dependent defense responses against *B.c.*, whilst upregulating ethylene emissions, which would tentatively help prevent an excessive proliferation of the fungus.

### Conclusions and perspectives

We demonstrated that plants experiencing WL+FR light display shoot and petiole elongation, typical shade avoidance traits accompanied by increased foliar susceptibility to *B.c.*. By performing a transcriptome analysis over a 30-h infection period, we found that supplemental FR dampens and delays the overall gene modulation in response to *B.c.* and especially JA-mediated responses in turn enhancing susceptibility in tomato. This phenomenon occurs via a reduced JA responsiveness possibly postponing *PI* gene induction and the associated JA-mediated plant immune responses. In addition, supplemental FR suppresses the classic ROS burst upon chitosan elicitation and increases ion leakage. The elevated ethylene emissions in WL+FR-pretreated plants reduce pathogen development and may prevent excessive pathogen proliferation in low R:FR light conditions. The data collectively provide a broad overview and understanding of supplemental FR-induced disease susceptibility. As we focused our work at the infection site, it is of great importance to assess the involvement of systemic signaling in the FR-induced susceptibility in tomato plants in future studies. The defense peptide systemin would be one candidate, but also mobile hormones could orchestrate systemic responses. Studies into this question could also aid in separating light and defense signaling with, that is mutagenization, grafting or local illumination methods to quantify the effect of light and defense as single parameters to better understand the molecular mechanisms of this complex tradeoff. Since tomato is genetically tractable, can be transformed and can also easily be used for grafting approaches, this species might be a highly suitable model system to study both local and systemic signaling in the growth-defense tradeoff. We anticipate this can aid further improvement of tomato genotypes for growth at high density.

## Materials and methods

### Plants growth conditions and light treatments

Tomato (*Solanum lycopersicum*) cv. Moneymaker (LA2706) seeds were obtained from the C. M. Rick Tomato Genetics Resource Center and propagated by the Horticulture and Product Physiology group at Wageningen University and Research as part of the LED it Be 50% consortium. Seeds were sown in wet vermiculite. After 10 d, seedlings were transferred into 9 × 9 cm pots with regular potting soil (Primasta soil, the Netherlands). Tomato plants were grown for 4 weeks after sowing in climate chambers (MD1400; Snijders, The Netherlands) in long day photoperiod (8 h dark/16 h light) at 150 µmol m^−2^ s^−1^ photosynthetically active radiation (PAR) using Philips GreenPower LED research modules (Signify B.V) white (WL, R:FR = 5.5) reaching a Phytochrome Stationary State (PSS) value of 0.8 (Sager et al., 1988). Temperature was set to 21°C and kept constant throughout day and night. For supplemental FR treatments, 4-week old plants were exposed for 5 d (starting at ZT = 3 on the first day) under WL supplemented with Philips GreenPower LED research modules far-red (FR; WL+FR; R:FR = 0.14, PSS value = 0.5, PAR = 150 µmol m^−2^ s^−1^). The top two lateral leaflets of the third leaf were used for all experiments. Light spectra used in this study were measured with a JAZ spectrophotometer (Ocean Optics Inc., UK) and are displayed in Supplemental Figure S1. Stem length was measured with a digital caliper. Petiole angles were measured from pictures with the ImageJ software. All measurements were calculated as Days 5 to 1.

### Leaf thickness measurements

Fresh leaf pieces from intact plants were cut and immediately incubated in 1–2 mL of Karnovski’s fixative solution (2.1% formaldehyde [v/v], 2.5% glutaraldehyde [v/v], and 0.1 M phosphate buffer [0.2 M NaH_2_PO_4_; 0.2 M Na_2_HPO_4_] at pH = 7). The samples were vacuum infiltrated for 10–15 min and slowly shaken at room temperature for 1 h before three washes with MilliQ water. Samples were dehydrated by adding 1–2 mL of a graded series of ethanol (30%, 50%, 70%, 90%, 96% [v/v]) replacing the ethanol every 30–60 min. Dehydrated leaf samples were embedded following the Technovit 7100 plastic embedding system and shaped into a trapezoid with until the leaf tissue was visible in the middle. Sections of 10 µm were made using a Leica Om-U3 microtome and mounted on microscope slides supplemented with a drop of MilliQ water and the slides were dried on a heating plate (80°C). The sections were stained with 0.5% toluidine blue (v/v) for 30–60 s and washed thoroughly with MilliQ water. Images were taken and leaf thickness was measured using an Olympus fluorescent microscope BX50-WI.

### Fungal growth conditions and detached leaflets bioassays

*Botrytis cinerea* B05.10 was maintained on half strength potato dextrose agar medium (PDA ½, BD Difco) and cultivated for 2 weeks under natural daylight conditions at room temperature. The spore suspension was prepared according to Van Wees et al. (2013), but diluted to a lower, final concentration of 1.5 × 10^5^ spores mL^−1^ in half strength potato dextrose broth (PDB ½, BD Difco) prior to the inoculation, since this concentration allowed for good lesion development and resolution in scoring disease symptoms on tomato leaves upon infection. Bioassays were performed on detached tomato leaflets previously treated in WL or WL+FR for 5 d. The leaflets were placed in square Petri dishes onto Whatman filter soaked with 6 mL of tap water to avoid dehydration. The bioassays took place at 3 p.m. The adaxial side of the leaflets was drop-inoculated three to six times with 5 µL of spore suspension. Plates were sealed with PARAFILM M and incubated for 3 d in their respective light treatment conditions (WL or WL+FR). Pictures were taken 3 d post inoculation (dpi) and lesion areas were measured using the ImageJ software with the image processing package Fiji (Schindelin et al., 2012).

### ROS measurements

Tomato plants (4-week-old) were exposed to either WL or WL+FR for 4 d. On Day 5, leaf discs (Ø 0.4 cm) originating from the third leaf of each plant were floated on deionized water for approximately 8 h in either WL or WL+FR corresponding to the light treatment conditions at room temperature without shaking. Each leaf disc was placed in each well of a white flat-bottomed 96-well plate (Greiner LUMITRAC 200) onto 180 µL of deionized water mixed with 20 µL of 10X reaction mix (200-µM Luminol L-012 and 10-µg mL^−1^ horseradish peroxidase) with a final concentration of 20-µM Luminol and 1 µg mL^−1^ of peroxidase. The luminescence was quantified by using a GloMax luminometer (Promega). The background noise was measured for 15 min prior to adding 1-µM flg22 or 1% chitosan (w/v) (final concentrations) on the leaf discs. The luminescence was measured for ∼1 h by recording 34 cycles of 100 s (Albert and Fürst, 2017).

### Ion leakage

Three leaf discs (Ø 0.6 cm) originating from the third oldest leaf of each plants were rinsed in deionized water to remove attached electrolytes leftover from the cutting. The conductivity was measured with an electrical conductivity meter after 3 h of incubation in 10 mL of 400-mM mannitol and again after 20 min at 95°C. The electrolyte loss was calculated as a percentage of the total amount of electrolytes in leaf tissue indicated by the value measured after boiling.

### Chemical treatments

Five days after the start of the WL or WL+FR pretreatment, tomato leaflets from the third leaf were detached and immediately dipped for 10 s in a 50-µM or 100-µM MeJA (Sigma-Aldrich) or mock solution (0.1% EtOH v/v) supplemented with 0.1% Tween-20 (v/v) then placed into separate sealed Petri dishes. When performed on whole plants, the third leaf was sprayed with 100-µM MeJA or a mock solution (0.1% Tween-20 [v/v]). Hormone treatments started at 10 a.m. (ZT = 3) in either WL or WL+FR. For gene expression experiments, leaf material was sampled for every condition at 0 min, 15 min, and 4 h after the start of the MeJA treatment. For bioassays, detached MeJA-treated tomato leaflets were inoculated in WL conditions with *B.c.* spores 4 h after MeJA application. The same procedures and experiments were performed by using the JA biosynthesis inhibitor Jarin-1 (50 µM; Meesters et al., 2014).

For ethylene-related experiments, tomato leaflets from the third leaf were detached and placed in square Petri dishes containing two discs of Whatman filter paper soaked with tap water. Petri dishes were placed in separate transparent glass desiccators. Control plates were placed in a desiccator of which the lid was slightly opened to allow for air circulation. Ethylene treatments were performed by either replacing tap water by 6 mL of a 10-µM ACC (ethylene precursor) solution or by injecting gaseous ethylene (10 p.p.m.) in the desiccator. Ethylene inhibitor treatments were performed by injecting 1-MCP (10 p.p.m.) in the desiccator. All treatments lasted for 1 h prior to inoculation with *B.c.* spores as previously described.

### Ethylene quantification by gas chromatography

Tomato leaflets were detached from WL- or WL+FR-pretreated plants, weighed, carefully rolled and inserted in a 1-mL syringe. After 30 min, the gas contained in the syringe was injected into a gas chromatography device (Syntech Spectras GC955) to determine ethylene levels.

### RNA isolation and gene expression analysis

Leaf discs treated with either JA or mock solution were sampled for RNA isolation (three or four biological replicates per treatment) and ground with silica beads for 1 min in a tissue lyser. Stem tissues were ground by hand with a ceramic mortar and pestle. Samples were supplemented with 300 µL of cell lysis buffer (2% SDS w/v, 68-mM sodium citrate, 132-mM citric acid, and 1-mM EDTA) and incubated for 5 min at room temperature prior to adding 100 μL of protein/DNA precipitation buffer (4 M NaCl, 16-mM sodium citrate, and 32-mM citric acid) followed by 10 min of incubation on ice. Samples were centrifuged for 15 min at 13,000 rpm, while the supernatant was transferred to a new tube (300 µL) and supplemented with an equal volume of ice cold isopropanol. All tubes were centrifuged for 5 min at 13,000 rpm, and the pellet was washed with 300 µL of 70% ethanol (v/v). RNA pellets were air dried for 5 min before elution in 30-µL RNAse-free water. cDNA synthesis was carried out using RevertAid H minus Reverse transcriptase (Thermo scientific). Gene expression analysis was performed by RT-qPCR in a Viia7PCR device with 5-µL reaction mix containing SyberGreen Supermix (Bio-Rad). Tomato actin was used as a reference gene and fold change in expression were calculated based on 2^(^^−^^ddCt)^ (Livak and Schmittgen, 2001). Primer sequences used are displayed in Supplemental Dataset S3.

### Statistical analyses

Statistical analysis was performed by either a one-way ANOVA followed by Tukey’s post hoc test (*P* < 0.05) or Student’s *t* test (*P* < 0.05).

### Accession numbers

The RNA sequencing materials from this article can be found in the NCBI GEO data libraries under the accession number GSE157831. https://www.ncbi.nlm.nih.gov/geo/query/acc.cgi?acc=GSE157831).

## Supplemental data

The following materials are available in the online version of this article.

**Supplemental methods.** Methods used for supplemental data as well as for the RNA-sequencing time series bioassay, library preparation, and sequencing data analysis.

**Supplemental Figure S1.** LED light spectra used in this study.

**Supplemental Figure S2.** Effect of supplemental FR on lamina area, lamina dry weight and specific leaf area.

**Supplemental Figure S3.** Bioassay on whole plants exposed to WL or WL+FR.

**Supplemental Figure S4.** qPCR on *B.c.* genomic DNA in infected leaf tissue and mycelium growth assay in vitro.

**Supplemental Figure S5.** qPCR on WL+FR-regulated genes throughout the 5-d WL and WL+FR light pretreatment.

**Supplemental Figure S6.** Heatmap corresponding to the log_2_FC for the 131 genes significant for the three-way interaction (light*infection*time) in response to *B.c.* infection in either WL or WL+FR-pretreated plants.

**Supplemental Data Set S1.** RNA-sequencing sample description (metadata).

**Supplemental Data Set S2.** GO enrichment based on ANOVA (one, two, or three factors).

**Supplemental Data Set S3.** Primers used for conventional qPCR and qPCR on genomic DNA.

**Supplemental Data Set S4.** RNA-sequencing raw read counts.

**Supplemental Data Set S5.** RNA-sequencing normalized read counts.

**Supplemental Data Set S6.** Correlation value between all samples.

**Supplemental Data Set S7.** Log_2_ fold change, *P*-value and adjusted *P*-value per gene, per comparison and per timepoint.

**Supplemental Data Set S8.** GO enrichment per timepoint and per comparison.

## Supplementary Material

kiab354_Supplementary_Data

## Notes

R.P., S.W., and S.C. designed the research; S.C. carried out the biological experiments; B.L.S. performed the transcriptome analysis with input from K.K.; L.L. performed the RT-qPCR shown in Supplemental Figure S5; S.C., R.P., and K.K. discussed the results; S.C. and B.L.S. designed and made the figures; S.C wrote the manuscript with contributions of R.P., S.W., K.K., and B.L.S.

The author responsible for distribution of materials integral to the findings presented in this article in accordance with the policy described in the Instructions for Authors (https://academic.oup.com/plphys/pages/General-Instructions) is: Ronald Pierik (r.pierik@uu.nl).
