# Supplementary material for: Mechanisms of far-red light-mediated dampening of defense against Botrytis cinerea in tomato leaves
Source: Plant Physiol. 2021 Jul 24;187(3):1250–66. doi: 10.1093/plphys/kiab354 (PMC8566310; doi:10.1093/plphys/kiab354)
Supplement: kiab354_Supplementary_Data [file kiab354_supplementary_data.zip › 20210610_Revise_SUPPLEMENTAL METHODS AND FIGURESFINAL.pdf]

Supplemental materials

Supplemental methods

Fungal growth assays *in vitro*

In vitro mycelium diameter measurements were performed by depositing a 5 µl droplet of 1.5 x 10^5 spores ml^{-1} solution onto PDA ½ plates. For biomass assays, a sterile mesh membrane was placed onto PDA ½ plates and soaked with 1 ml of 1.5 x 10^5 spores ml^{-1} suspension. For both experiments, plates were sealed and incubated for three days in standard growth chamber light at 20°C in long day conditions (16 hr day, 8 hr night; ~180 µmol m^{-2} s^{-1} PAR; R:FR = 1.8) with or without additional FR light (~730 nm, Phillips; R:FR = 0,2). The newly-grown mycelium diameter was measured by using a digital caliper. For biomass assays, the membranes were removed from the plates, dried and weighed. The mycelium biomass was calculated as the weight difference between day 3 and day 0.

Bioassay and harvest for RNA-sequencing time series

Four-week-old tomato plants were pretreated in WL or WL+FR respectively and drop-inoculated 10 hours after dawn with a 1.5 x 10^5 spores ml^{-1} solution or a mock solution only containing PDB ½ medium. Each leaflet was placed in an individual round petri dish (9 cm) and the infection took place in WL conditions. Five leaf discs (ø0.5 cm) were collected at 0, 6, 12, 18, 24, and 30 hours post inoculation (hpi). A total of 95 samples corresponding to two treatment light conditions (WL and WL+FR), two infection conditions (*B. cinerea* and mock), following six time points with three to four replicates per samples were collected. Samples were snap-frozen in liquid nitrogen and kept at -80 °C until RNA isolation.

RNA-sequencing library preparation

We prepared non-strand specific library according to Townsley et al. (2015) with modifications. Harvested leaf discs were finely ground with a tissue lyser (MM 400, Retsch) and resuspended in 400 µl of lysis/binding buffer (LBB; 100 mM Tris-HCl pH = 8, 1 M LiCl, 10 mM EDTA, 1% SDS, 5 mM DTT, 15 µl ml^{-1} Antifoam A). LBB was supplemented with 5 µl ml^{-1} β-mercaptoethanol right before use. The samples were bead beaten again for 2 x 30 sec and incubated at room temperature for 10 min before spinning 10 min at 13000 rpm. Total mRNA was isolated from 200 µl of lysate. 1 µl of 12.5 µM biotin-20nt-dT oligo primer (5’-

Biotin-ACAGGACATTCGTCGCTTCCTTTTTTTTTTTTTTTTTTT-3') was added to the lysate and incubated for 10 min at room temperature before addition of 20 µl of washed streptavidin coated beads (New England Biolabs). The samples were placed on a Magwell® 96 well magnetic separator and the supernatant was discarded. The pellets were washed in 200 µl of Washing buffer A (WBA) containing 10 mM Tris-HCl, 150 mM LiCl, 1 mM EDTA and 0.1% SDS then with 200 µl of washing buffer B (WBB) corresponding to WBA without SDS. The bead pellets were washed one last time in low salt buffer (LSB) containing 20 mM Tris-HCl, 150 mM NaCl and 1 mM EDTA. The process was repeated another time to improve RNA purity. RNA fragmentation and cDNA priming were performed by mixing 1.5 µl of 5 X Thermo scientific RT buffer, 0.5 µl random primers (Invitrogen®) and 8 µl of RNA. The mixed reagents were placed in a thermocycler (25°C – 1 sec ; 94°C – 1.5 min ; 4°C – 5 min ; 4°C – hold). 5 µl of fragmented RNAs was added to the following reagents mixture 1.5 µl of 5X Thermo scientific RT buffer, 0.1 M DDT, 1 µl H_2O, 0.5 µl 25 mM dNTPs and 0.5 µl RevertAid Reverse Transcriptase enzyme (Thermo scientific). The total reaction volume was 15 µl and was incubated in a thermocycler for reverse transcription of the first cDNA strand (25°C – 10 min, 42°C – 50 min, 50°C – 10 min 70°C – 10 min and 4°C - hold). The second strand synthesis was performed as follows with 1.5 µl H_2O ; 0.4 µl 25nM dNTPs ; 1 µl PolI (Thermo scientific) ; 0.1 µl RNAseH (Biolabs) ; 0.4 µl End repair module (T4 Pol + PNK mix) ; 0.2 µl Taq polymerase and 1.4 µl End repair buffer. The total reaction volume of 10 µl was placed in a thermocycler following the program: 16°C – 20 min ; 20°C – 20 min ; 72°C – 20 min ; 4°C – hold. Non-strand specific universal primer adapters were prepared by mixing 8 µl of 100 µM PE1-lig primer (5'-CACTCTTTCCCTACACGACGCTCTTTCCGATCT-3') and 8 µl of 100 µM 5'phosphorylated ILL-lig primer (5'-P-GATCGGAAGAGCACACGTCTGAACTCCAGTCAC-3') in 784 µl of H_2O. The total volume was equally divided over a 8-well strip and followed the PCR program 94°C – 1 min ; (94°C – 10 sec) x 60 -1°C per cycle ; 20°C – 1 min ; 4°C – hold). cDNAs were cleaned up by a 5 min incubation at room temperature with 30 µl of XP Ampure beads (Beckman counter) and washed twice with 200 µl of 80% ethanol. The bead pellets were put to dry at room temperature on magnetic separator. Non-strand specific adapters were ligated onto cDNAs by adding 3 µl of annealed 1 µM universal primers on dry cDNA pellets. Once eluted, the cDNA/universal primers mix was supplemented with 7 µl the following reaction mix : 1.75 µl H2O ; 5 µl 2X Rapid T4 ligase buffer ; 0.25 µl DNA ligase (Biolabs) and let to sit for 15 min. 10 µl of 50 mM EDTA and 25 µl of ABR resuspension buffer (15% PEG 8000 ; 2.5 M NaCl) were added to the mix and let to incubate at room temperature for 5 min before placing it on

the magnetic tray. The bead pellets were washed twice with 200 µl of 80% ethanol without resuspension. The samples were eluted in 22 µl of 10 mM Tris-HCl and enriched by PCR. 5 µl of each cDNA sample coupled with the universal adapters was supplemented with 0.5 µl of the appropriate indexed enrichment oligo (see annex for enrichment oligo sequences) and 4.5 µl of the following mix : 2 µl 5X Phusion HF Buffer, 1.3 µl H_2O, 0.5 µl PE1 primer (5'-AATGATACGGCACCACCGAGATCTACACTCTTTCCCTACACGAC-GCTCTTCCGATCT-3'), 0.5 µl 8 µM each S1 and S2 primers (S1: 5'-AATGATACGGCGACCACCGA-3' and S2: 5'-CAAGCAGAAGACGGCATACGA-3' respectively), 0.1 µl of 25 mM dNTPs and 0.1 µl Phusion polymerase. The mix followed the thermocycler program: 98°C - 30 sec, (98°C - 10 sec, 65°C - 30 sec, 72°C - 30 sec) x 11 cycles; 72°C - 5 min and hold at 10°C. The non-strand specific libraries were cleaned one last time to select for 350 bp molecules. The libraries were size-selected with 1.1 volumes of Ampure XP beads per samples and incubated at room temperature for 5 min before washing twice with 80% ethanol on magnetic rack. The final product was eluted in 10 µl 10 mM Tris-HCl.

RNA Sequencing and data analysis

All cDNA libraries were sequenced using 1 x 75 pb high output Illumina NEXTseq500. All 95 libraries were pooled as one sample and two sequencing runs were necessary to achieve minimally 3.000.000 mapped reads per library. The quality of the libraries was controlled with FastQC v0.10.1 (available online at: http://www.bioinformatics.babraham.ac.uk/projects/fastqc). Sequencing reads were mapped against the tomato reference transcriptome version SL3.0 with ITAG3.20 annotations (https://solgenomics.net/organism/Solanum_lycopersicum/genome) using STAR v2.4.2a (Dobin et al., 2013) and counted using HTSeq-Count 0.6.1p1 (Anders et al., 2015) (Supplemental dataset S3). Reads were normalized by their size factor via quantile normalization using the R package DESeq v1.18.0 (Supplemental dataset S4). Principal coordinate analyses were done in R 3.6.0 (R Core team, 2013). Differentially expressed genes were determined per timepoint using an ANOVA, with the model: gene expression ~ infection * light treatment + replication. "Gene expression" is calculated per gene as the log2 ratio of each observation of a gene with the mean expression of that same gene over all samples. "Infection" is the mock or *B. cinerea* treatment, and "light" is the control (WL) of pre-treatment with FR light. To calculate the differences between the four different treatments, TukeyHSD was used. All calculations were done in R 3.6.0 (R Core team, 2013). The p-values and effects were recorded for further investigation (Supplemental dataset S5 and S6). An FDR adjusted p-

value (BH method (Benjamini and Hochberg, 1995) of the p.adjust() function) was used to determine the number of genes significantly up or down regulated per timepoint. A p-value < 0.001 was used to select the differentially expressed genes for GO term enrichment. GO enrichment was done in R 3.6.0 (R Core team, 2013), using the hypergeometric test on the differentially expressed genes per time point. The GO term identifiers were obtained from solgenomics.net based on the tomato reference genome version SL3.0 with ITAG3.20 annotations. Clustering behind the heatmaps for GO-enrichments and DEGs were performed using heatmapper.ca with default settings (Supplemental dataset S7 and S8).

qPCR on *B. cinerea* genomic DNA

B. cinerea genomic DNA was extracted from 3 infected leaf discs per samples, ground and incubated at 65°C for 45 min in 250 µl of DNA extraction buffer (EDTA 25 mM, Tris-HCl 250 mM, NaCl 250 mM and SDS 1%). The samples were supplemented with 250 µl of phenol-chloroform (PCI) and centrifuged for 7 min at full speed. The upper phase was transferred into a new tube and 250 µl of ice-cold isopropanol were added. Precipitated DNA was pelleted by spinning 7 min at full speed prior to 2 washes in EtOH 70%. The air-dried pellets were eluted in 100 µl of MilliQ water. All DNA samples were diluted to 5 ng μl^{-1} and 15 ng μl^{-1}. The qPCR was performed with SybrGreen Supermix (Bio-Rad) and specific primers (see supplemental data S1) in a Viia7 PCR machine. Amplification data were analyzed using the $2^{-\Delta\Delta Ct}$ method (Livak and Schmittgen, 2001).

References

Anders S, Pyl PT, Huber W (2015) HTSeq-A Python framework to work with high-throughput sequencing data. Bioinformatics **31**: 166–169

Benjamini Y, Hochberg Y (1995) Controlling the False Discovery Rate: A Practical and Powerful Approach to Multiple Testing. J R Stat Soc Ser B **57**: 289–300

Dobin A, Davis CA, Schlesinger F, Drenkow J, Zaleski C, Jha S, Batut P, Chaisson M, Gingeras TR (2013) STAR: Ultrafast universal RNA-seq aligner. Bioinformatics **29**: 15–21

Livak KJ, Schmittgen TD (2001) Analysis of relative gene expression data using real-time quantitative PCR and the 2-ΔΔCT method. Methods **25**: 402–408

Townsley BT, Covington MF, Ichihashi Y, Zumstein K, Sinha NR (2015) BrAD-seq:

Breath Adapter Directional sequencing: A streamlined, ultra-simple and fast library preparation protocol for strand specific mRNA library construction. Front Plant Sci **6**: 1–11

Supplemental figures

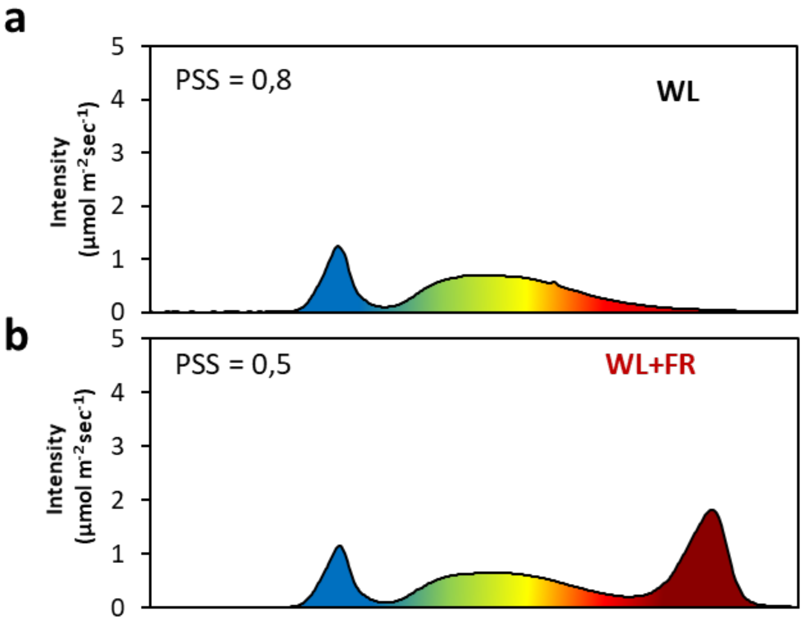

Figure S1: LED light spectra used in this study. Light (pre)treatments were either performed under white LEDs (WL) or WL supplemented with far-red LEDs (WL+FR). Spectra were measured using a JAZ spectrophotometer (Ocean Optics Inc., UK).

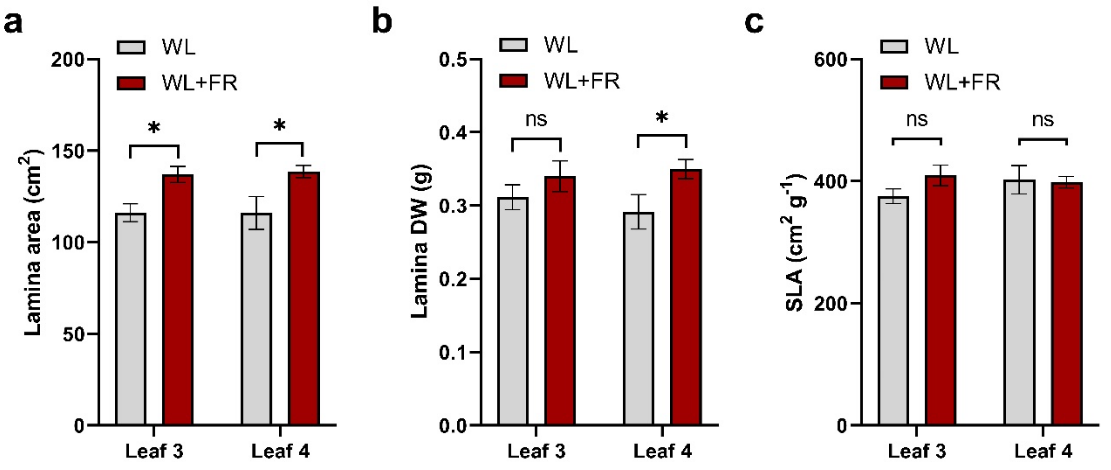

Figure S2: Supplemental FR has only minor effects of leaf area and dry weight. (**a**) Lamina area and (**b**) dry weight measurements on the third and fourth oldest leaf after five days of either WL or WL+FR exposure on whole tomato plants. Both parameters were used to determine the (**c**) specific leaf area of both leaves under the two light treatments. Data represent mean ± SEM, n = 8. Asterisks represents significant differences according to Student’s t-test ($p < 0.05$). ns refers to a non-significant difference.

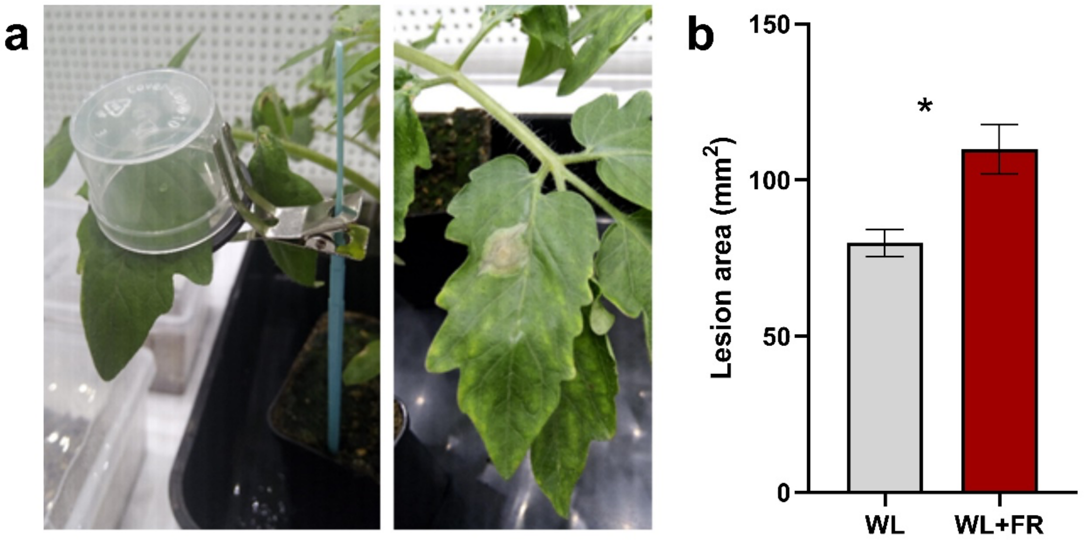

Figure S3: FR-enriched light increases susceptibility in whole plants. (a) Local high humidity was achieved by clipping a polyethylene terephthalate (PET) cup with rubber ring on the leaf to avoid wounding. **(b)** Disease rating on intact tomato leaflets after five days of WL and WL+FR light treatments inoculated with *B. cinerea* spores. Lesions were measured at 3 dpi with the ImageJ software. Data show mean ± SEM and asterisk represents significant difference according to Student's t-test ($p < 0.05$), n = 7 - 8 plants per treatment.

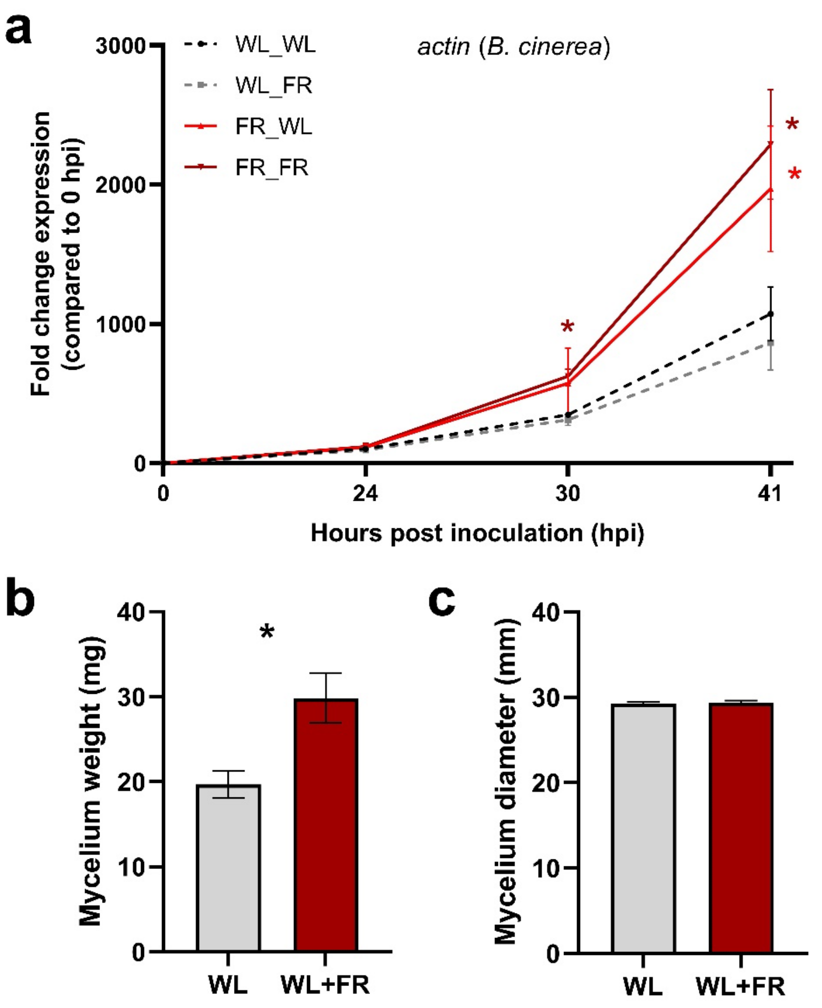

Figure S4: Supplemental FR affects *Botrytis* growth *in planta* and *in vitro*. (a) Quantitative real-time PCR (qPCR) on genomic DNA of *B. cinerea* (actin) relative to a tomato reference gene (elongation factor alpha) in WL or WL+FR-treated plant tissue after 0, 24, 30 and 41 hpi (hours post inoculation). The WL or WL+FR pretreatment was performed on whole plants prior to inoculation on detached leaflets. Asterisks represent significant differences according to a Student's t-test comparing each treatment to WL_WL conditions per timepoint ($p < 0.05$), n = 3. **(b)** Newly grown *B. cinerea* mycelium biomass and **(c)** diameter after three days of incubation in either WL or WL+FR conditions. Data represent mean +SEM. Asterisks represent statistical difference according to a Student's t-test compared to WL control conditions ($p < 0.05$), n = 10.

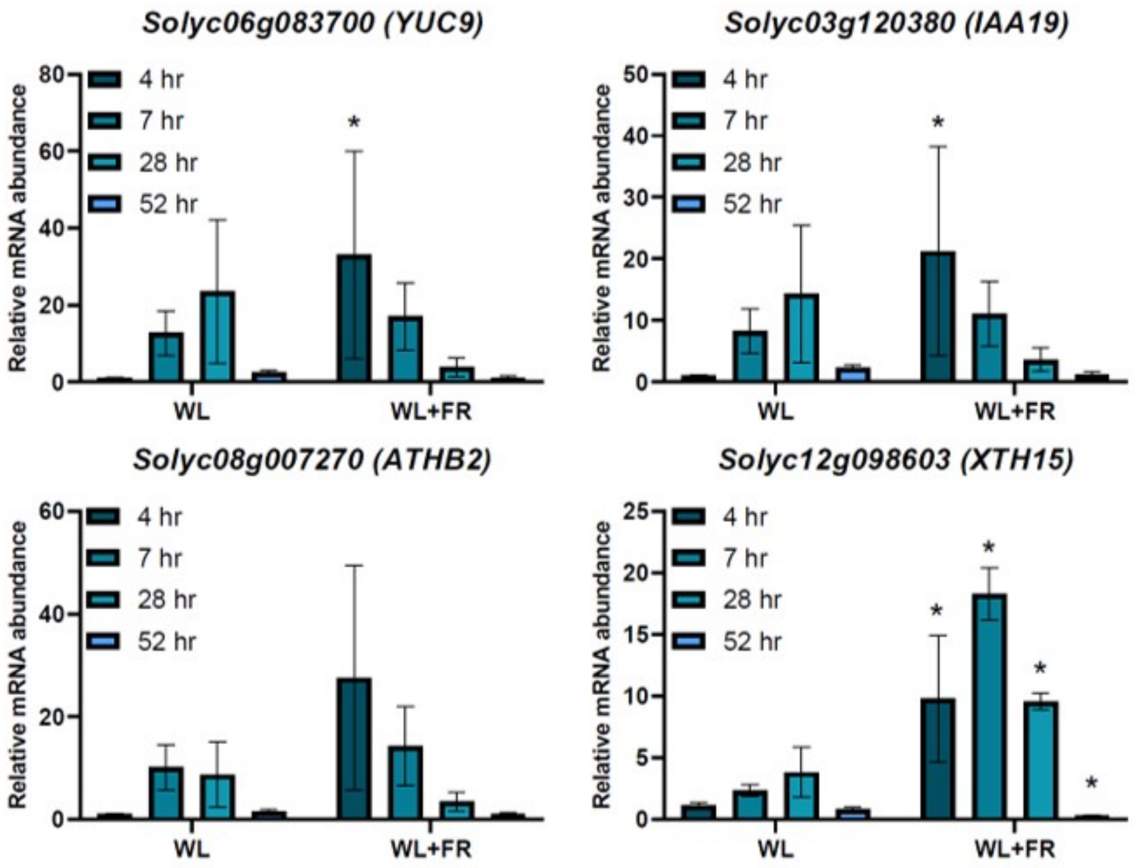

Figure S5: WL+FR-pretreatment duration affects the expression of FR-responsive genes. Quantitative real-time PCR (q-RT-PCR) on FR-regulated genes relative to a reference gene (Actin) in WL or WL+FR-treated stem tissue originating from the third internode between the second and third oldest leaf after 4, 7, 28, 52 hours after the start of the WL+FR pretreatment. pretreatment was performed on whole plants. Values are relative to the mean of the WL-treated samples at 4h. Asterisks represent significant differences according to a Student's t-test comparing WL+FR-treated samples to WL conditions for each timepoint ($p < 0.05$), n = 5.

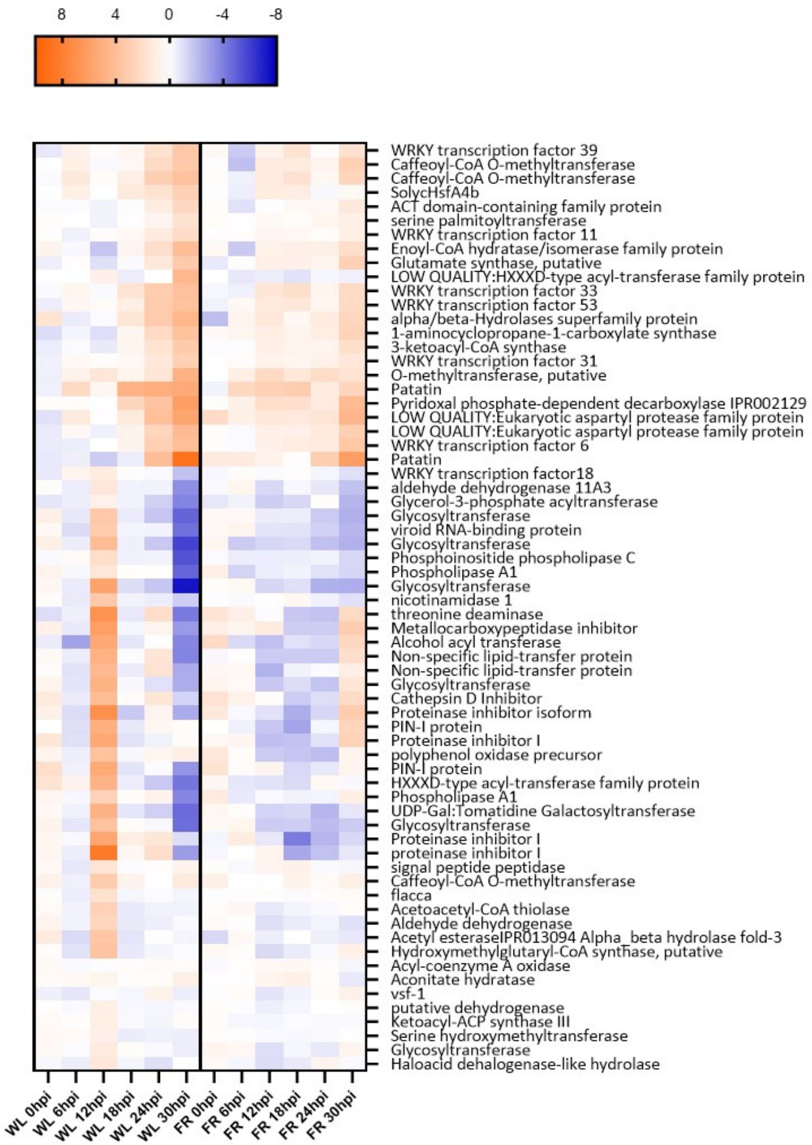
8
4
0
-4
-8
WRKY transcription factor 39
Caffeoyl-CoA O-methyltransferase
Caffeoyl-CoA O-methyltransferase
SolycHsfA4b
ACT domain-containing family protein
serine palmitoyltransferase
WRKY transcription factor 11
Enoyl-CoA hydratase/isomerase family protein
Glutamate synthase, putative
LOW QUALITY:HXXXD-type acyl-transferase family protein
WRKY transcription factor 33
WRKY transcription factor 53
alpha/beta-Hydrolases superfamily protein
1-aminocyclopropane-1-carboxylate synthase
3-ketoacyl-CoA synthase
WRKY transcription factor 31
O-methyltransferase, putative
Patatin
Pyridoxal phosphate-dependent decarboxylase IPR002129
LOW QUALITY:Eukaryotic aspartyl protease family protein
LOW QUALITY:Eukaryotic aspartyl protease family protein
WRKY transcription factor 6
Patatin
WRKY transcription factor18
aldehyde dehydrogenase 11A3
Glycerol-3-phosphate acyltransferase
Glycosyltransferase
viroid RNA-binding protein
Glycosyltransferase
Phosphoinositide phospholipase C
Phospholipase A1
Glycosyltransferase
nicotinamidase 1
threonine deaminase
Metallocarboxypeptidase inhibitor
Alcohol acyl transferase
Non-specific lipid-transfer protein
Non-specific lipid-transfer protein
Glycosyltransferase
Cathepsin D Inhibitor
Proteinase inhibitor isoform
PIN-I protein
Proteinase inhibitor I
polyphenol oxidase precursor
PIN-I protein
HXXXD-type acyl-transferase family protein
Phospholipase A1
UDP-Gal:Tomatidine Galactosyltransferase
Glycosyltransferase
Proteinase inhibitor I
proteinase inhibitor I
signal peptide peptidase
Caffeoyl-CoA O-methyltransferase
flacca
Acetoacetyl-CoA thiolase
Aldehyde dehydrogenase
Acetyl esteraseIPR013094 Alpha_beta hydrolase fold-3
Hydroxymethylglutaryl-CoA synthase, putative
Acyl-coenzyme A oxidase
Aconitate hydratase
vsf-1
putative dehydrogenase
Ketoacyl-ACP synthase III
Serine hydroxymethyltransferase
Glycosyltransferase
Haloacid dehalogenase-like hydrolase
WL 0hpi
WL 6hpi
WL 12hpi
WL 18hpi
WL 24hpi
WL 30hpi
FR 0hpi
FR 6hpi
FR 12hpi
FR 18hpi
FR 24hpi
FR 30hpi

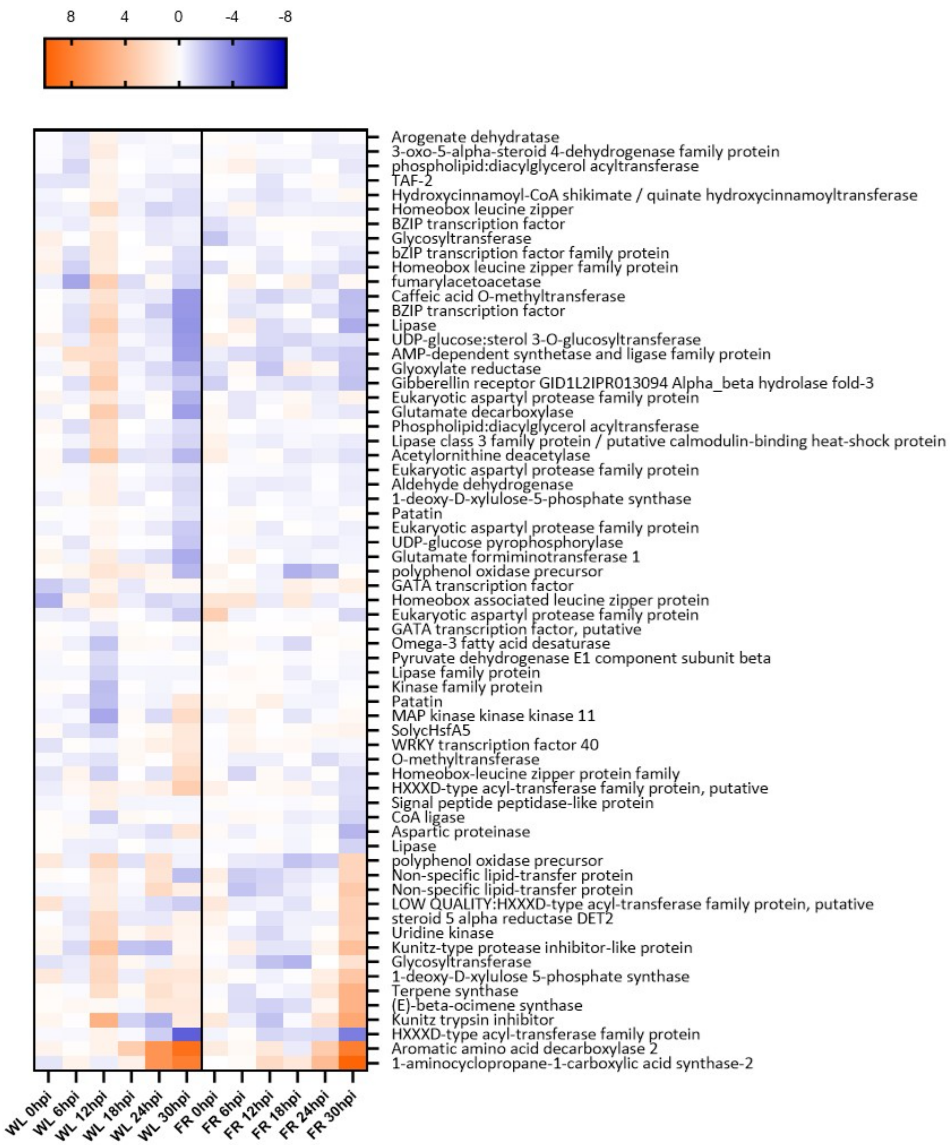
8
4
0
-4
-8
Arogenate dehydratase
3-oxo-5-alpha-steroid 4-dehydrogenase family protein
phospholipid:diacylglycerol acyltransferase
TAF-2
Hydroxycinnamoyl-CoA shikimate / quinate hydroxycinnamoyltransferase
Homeobox leucine zipper
BZIP transcription factor
Glycosyltransferase
bZIP transcription factor family protein
Homeobox leucine zipper family protein
fumarylacetoacetase
Caffeic acid O-methyltransferase
BZIP transcription factor
Lipase
UDP-glucose:sterol 3-O-glucosyltransferase
AMP-dependent synthetase and ligase family protein
Glyoxylate reductase
Gibberellin receptor GID1L2IPR013094 Alpha_beta hydrolase fold-3
Eukaryotic aspartyl protease family protein
Glutamate decarboxylase
Phospholipid:diacylglycerol acyltransferase
Lipase class 3 family protein / putative calmodulin-binding heat-shock protein
Acetylornithine deacetylase
Eukaryotic aspartyl protease family protein
Aldehyde dehydrogenase
1-deoxy-D-xylulose-5-phosphate synthase
Patatin
Eukaryotic aspartyl protease family protein
UDP-glucose pyrophosphorylase
Glutamate formiminotransferase 1
polyphenol oxidase precursor
GATA transcription factor
Homeobox associated leucine zipper protein
Eukaryotic aspartyl protease family protein
GATA transcription factor, putative
Omega-3 fatty acid desaturase
Pyruvate dehydrogenase E1 component subunit beta
Lipase family protein
Kinase family protein
Patatin
MAP kinase kinase kinase 11
SolycHsfA5
WRKY transcription factor 40
O-methyltransferase
Homeobox-leucine zipper protein family
HXXXD-type acyl-transferase family protein, putative
Signal peptide peptidase-like protein
CoA ligase
Aspartic proteinase
Lipase
polyphenol oxidase precursor
Non-specific lipid-transfer protein
Non-specific lipid-transfer protein
LOW QUALITY:HXXXD-type acyl-transferase family protein, putative
steroid 5 alpha reductase DET2
Uridine kinase
Kunitz-type protease inhibitor-like protein
Glycosyltransferase
1-deoxy-D-xylulose 5-phosphate synthase
Terpene synthase
(E)-beta-ocimene synthase
Kunitz trypsin inhibitor
HXXXD-type acyl-transferase family protein
Aromatic amino acid decarboxylase 2
1-aminocyclopropane-1-carboxylic acid synthase-2
WL 0hpi
WL 6hpi
WL 12hpi
WL 18hpi
WL 24hpi
WL 30hpi
FR 0hpi
FR 6hpi
FR 12hpi
FR 18hpi
FR 24hpi
FR 30hpi

Figure S6: Dynamics of expression of 3-way ANOVA light*infection*time. Heatmap corresponding to the log_2FC for the 131 genes significant for the 3-way interaction in response to *B. cinerea* infection after a 5-day in WL and WL+FR pretreatment and inoculation in WL at 0, 6, 12, 18, 24 and 30 hpi (hours post inoculation). Orange and blue colors represent the significantly up and downregulated genes, respectively.
